# Depletion of circulating blood NOS3 increases severity of myocardial infarction and left ventricular dysfunction

**DOI:** 10.1007/s00395-013-0398-1

**Published:** 2013-12-18

**Authors:** Marc W. Merx, Simone Gorressen, Annette M. van de Sandt, Miriam M. Cortese-Krott, Jan Ohlig, Manuel Stern, Tienush Rassaf, Axel Gödecke, Mark T. Gladwin, Malte Kelm

**Affiliations:** 1 Division of Cardiology, Pneumology and Angiology, Department of Medicine, University Hospital Düsseldorf, Moorenstrasse 5, 40225 Düsseldorf, Germany; 2 Department of Cardiovascular Physiology, Heinrich-Heine-University, Düsseldorf, Germany; 3 Vascular Medicine Institute, University of Pittsburgh, Pittsburgh, PA USA; 4 Division of Pulmonary, Allergy and Critical Care Medicine, Department of Medicine, University of Pittsburgh School of Medicine, Pittsburgh, PA USA

**Keywords:** Nitric oxide, Myocardial ischemia/reperfusion, Circulating NOS3

## Abstract

Nitric oxide (NO) derived from endothelial NO synthase (NOS3) plays a central role in myocardial ischemia/reperfusion (I/R)-injury. Subsets of circulating blood cells, including red blood cells (RBCs), carry a NOS3 and contribute to blood pressure regulation and RBC nitrite/nitrate formation. We hypothesized that the circulating blood born NOS3 also modulates the severity of myocardial infarction in disease models. We cross-transplanted bone marrow in wild-type and NOS3^−/−^ mice with wild-type mice, producing chimeras expressing NOS3 only in vascular endothelium (BC−/EC+) or in both blood cells and vascular endothelium (BC+/EC+). After 60-min closed-chest coronary occlusion followed by 24 h reperfusion, cardiac function, infarct size (IS), NO_x_ levels, RBCs NO formation, RBC deformability, and vascular reactivity were assessed. At baseline, BC−/EC+ chimera had lower nitrite levels in blood plasma (BC−/EC+: 2.13 ± 0.27 μM vs. BC+/EC+ 3.17 ± 0.29 μM; *p < 0.05), reduced DAF FM associated fluorescence within RBCs (BC−/EC+: 538.4 ± 12.8 mean fluorescence intensity (MFI) vs. BC+/EC+: 619.6 ± 6.9 MFI; ***p < 0.001) and impaired erythrocyte deformability (BC−/EC+: 0.33 ± 0.01 elongation index (EI) vs. BC+/EC+: 0.36 ± 0.06 EI; *p < 0.05), while vascular reactivity remained unaffected. Area at risk did not differ, but infarct size was higher in BC−/EC+ (BC−/EC+: 26 ± 3 %; BC+/EC+: 14 ± 2 %; **p < 0.01), resulting in decreased ejection fraction (BC−/EC+ 46 ± 2 % vs. BC+/EC+: 52 ± 2 %; *p < 0.05) and increased end-systolic volume. Application of the NOS inhibitor *S*-ethylisothiourea hydrobromide was associated with larger infarct size in BC+/EC+, whereas infarct size in BC−/EC+ mice remained unaffected. Reduced infarct size, preserved cardiac function, NO levels in RBC and RBC deformability suggest a modulating role of circulating NOS3 in an acute model of myocardial I/R in chimeric mice.

**Electronic supplementary material:**

The online version of this article (doi:10.1007/s00395-013-0398-1) contains supplementary material, which is available to authorized users.

## Introduction

NO derived from the endothelial NO synthase (NOS3) regulates coronary blood flow, evokes positive inotropic and lusitropic effects, improves myocardial relaxation and optimizes cardiac performance [41]. NO participates in the regulation of myocardial metabolism [30]. It reduces the consumption of oxygen and the inotropic effect of catecholamines by muscarinic, cholinergic, and beta-adrenergic receptor stimulation [1, 43].

During myocardial ischemia and reperfusion (I/R), NO exerts a cardioprotective role by a variety of mechanisms [15], e.g., it regulates mitochondrial respiration, thereby improving myocardial oxygenation [51]. Endogenous NO contributes to hibernation via reducing oxygen consumption and preserving calcium sensitivity and contractile function [15]. NO inactivates caspases by nitrosation and thus decreases myocyte apoptosis [15]. Whereas an increase in cardiac interstitial NO production could be observed during early I/R [29], which is in part derived from activated NOS isoforms [11], NO formation drops during ongoing I/R. In this acute phase, endothelium becomes dysfunctional, leukocyte adhesion increases and neutrophils migrate into the reperfused tissue. NOS3 knockout mice exhibit enlarged infarct sizes [20], while infarct size after I/R is reduced in animals with NOS3 overexpression [17, 44], suggesting a cardioprotective role for NOS3-derived NO in the setting of I/R.

NOS3 is not only expressed in the vascular endothelium but also in blood cells including B- and T-lymphocytes [42], eosinophils [50], and in red blood cells (RBCs) [5, 24]. RBCs are the most abundant blood cell population carrying a NOS3 and represent the major storage compartment of circulating NO metabolites [7, 39]. Red cell NOS3-dependent NO production alters the functional characteristics of the erythrocyte, including erythrocyte deformability, platelet activity and vascular tone [5, 24, 47]. Thus, in addition to the vascular endothelium, the RBCs are another source of vascular NOS-dependent NO production and contribute to the circulating NO pool [5, 24]. In addition, RBCs have “shuttle properties” and are able to accumulate and transport NO metabolites such as nitrite [9]. Application of RBCs with subsequent increase in NO release reduced the extent of irreversible myocardial tissue damage in isolated hearts [48].

We, therefore, hypothesized that circulating NOS3 decreases infarct size and subsequently preserves left ventricular function following myocardial I/R injury. To selectively assess infarct size in the absence or presence of circulating NOS3, we created chimera mice lacking or carrying blood cell NOS3 by transplanting bone marrow from NOS3^−/−^ mice or wild type (WT) into WT mice, and analyzed infarct size after 60-min closed-chest coronary occlusion followed by 24 h of reperfusion.

## Methods

### Animals

Male C57BL/6 wild-type (WT) and NOS3^−/−^ mice (endothelial nitric oxide synthase) (C57BL/6.129/Ola-eNOStm) [10] were kept according to federal regulations. All studies were approved by the state animal welfare commission. Mice ranged in body weight from 20 to 25 g and in age from 8 to 10 weeks for bone marrow transplantation.

### Chimeras (irradiation and bone marrow transplantation)

To analyze the effects of the lack of NOS3 in blood cells in an acute model of myocardial I/R, we transplanted bone marrow from WT and NOS3^−/−^ mice, producing chimeras which either do (BC+/EC+) or do not carry NOS3 in blood cells (BC−/EC+) as described previously [47] (See Online Resource 2 for detailed information).

### Blood collection, RBC preparation and loading with DAF-FM

Blood was obtained from mice via heart puncture, anticoagulated with heparin and processed within 2 h. For loading with DAF-FM diacetate, whole blood was diluted 1:500 to a final concentration of ~1.2 × 10^4^ RBC/μl in cold phosphate buffered solution (PBS) as previously described [4, 5]. In brief, RBCs were loaded with 10 μM DAF-FM diacetate for 30 min at room temperature in the dark, or left untreated, washed in PBS and analyzed for DAF FM-associated fluorescence in a FACS Canto II (BD Biosciences) flow cytometer. For NOS inhibition, RBC suspensions were pre-incubated for 30 min with 3 mM L-NAME (L-N^G^-nitroarginine methyl ester). The method was validated for detection of NO-related species in RBC by applying a multilevel analytical approach and separating the reaction products with RP-HPLC or LC/MS/MS, as described in [4, 5] (See Online Resource 2 for detailed information).

### Measurement of nitrite/nitrate in plasma, heart tissue and aorta

Blood samples were collected by intra-cardiac puncture at baseline, after 5 min and 24 h of myocardial reperfusion. Blood and tissue samples were prepared for determination of nitrate and nitrite as previously described [12, 23, 38, 40] (See Online Resource 5 and 6 for detailed information).

### Measurement of RBC deformability (ektacytometry)

Blood was drawn via heart puncture and collected in a heparinized tube for the measurement of RBC deformability. RBC deformability was measured by ektacytometry by the Laser-assisted optical rotational cell analyzer (LORCA, R&R Mechatronics) according to the manufacturer’s instructions as previously described [16, 21]. RBC deformability was expressed by the elongation index (EI), which was calculated from the elliptical RBC diffraction pattern as EI = (*L* − *W*)/(*L* + *W*), where *L* and *W* are the length and width of the diffraction pattern, respectively (See Online Resource 9 for detailed information).

### Langendorff setup

For isolated heart measurements, murine hearts were explanted at baseline (6 weeks after bone marrow transplantation), and mounted with retrograde perfusion at 100 mmHg constant pressure with modified Krebs–Henseleit buffer in an isolated heart apparatus (Hugo Sachs Elektronik), as previously described [31–33, 46] (See Online Resource 2 for detailed information).

### Gel electrophoresis and western blot analysis

Mouse heart, mouse aorta and human endothelial cells were lysed with RIPA lysis buffer containing protease inhibitor cocktail (Roche Applied Science), as previously described [5, 47]. Total protein concentration was determined by the Lowry assay (DC Protein Assay, Bio-Rad). For gel electrophoresis, 80 μg heart lysates, 20 μg aortic lysates, or human umbilical endothelial cell lysate were loaded in 4–12 % Bis–Tris gel. For western blot analysis, proteins were transferred onto polyvinylidene fluoride membrane Hybond P (Amersham Biosciences, Munich, Germany). A pre-stained protein ladder (PageRuler Plus, Fermentas Life Science) was loaded into the gel to control for transfer efficiency. The membrane was blocked with 5 % nonfat dry milk (Bio-Rad) in TBS (10 mM Tris, 100 mM NaCl), incubated with a mouse anti-human anti-eNOS antiserum (overnight 4 °C 1:500) (BD Bioscience) diluted (1 h RT 1:1,000) in T-TBS (0.1 % Tween in TBS), washed for 30 min in T-TBS, and then incubated with HRP-conjugated goat anti-mouse antibody (1:5,000) from (BD Bio science).

### Isometric force measurements in aortic rings

Thoracic aorta was removed as previously described at baseline (6 weeks after bone marrow transplantation) [45, 46]. Aortic rings were placed in an organ bath (Model Graz, Type 846, Hugo Sachs), under 1 g of tension, and bathed in 2 mL of Krebs buffer constantly gassed with 95 % O_2_/5 % CO_2_ at 37 °C. After equilibration phase (90 min), tissues were exposed to potassium chloride (80 nM) and subsequently phenylephrine (1 μM) to achieve maximal contraction. Afterwards relaxation response curves to increasing concentrations of acetylcholine (1 nM–10 μM) or to increasing concentrations of the NO donor sodium nitroprusside (SNP) (0.001–10 μM) were constructed. Contractility response to increasing concentrations of phenylephrine (1 nM–10 μM) was measured.

### Myocardial ischemia and reperfusion protocol

A closed-chest model of myocardial I/R was utilized 6 week after bone marrow transplantation to reduce surgical trauma and consequent inflammatory reaction following I/R as compared to open-chest model [36]. At 3-day post-instrumentation myocardial ischemia was induced for 60 min of ischemia followed by 24 h of reperfusion (See Online Resource 2 for detailed information).

### Assessment of infarct size (IS)

After 24 h of reperfusion, the animals were killed and heart was excised, rinsed in 0.9 % normal saline, left anterior descending artery (LAD) was re-occluded in the same location and 1 % Evans Blue dye was injected into the aortic root to delineate the area at risk (AAR) from not-at-risk myocardium, as published recently [14] (See Online Resource 2 for detailed information).

### Echocardiography

Cardiac images were acquired using a Vevo 2100 high-resolution ultrasound scanner with 18–38 MHz linear transducer (VisualSonics Inc.). Echocardiography was performed as previously described [26]. Left ventricular (LV) end-systolic (ESV), end-diastolic volumes (EDV), LV ejection fraction (EF), cardiac output (CO) and stroke volume (SV) were calculated (See Online Resource 8 for detailed information).

### ETU treatment

A subgroup of animals received *S*-ethylisothiourea hydrobromide (ETU) to achieve systemic NOS inhibition during ischemia and the first 5 min of reperfusion. During whole ischemia (60 min) and the first 5 min of reperfusion, ETU was administered at (0.245 μg/μl/min; i.p.) [46]. After 24 h of reperfusion, LV function was measured via echocardiography and infarct size was measured via triphenyltetrazoliumchlorid (TTC) staining in this subgroup.

### Statistical analysis

The results are given as mean ± standard error of the mean (SEM). For repeated measurements, data were analyzed by two-way ANOVA followed by Bonferroni’s post hoc test. Where indicated, an unpaired Student’s *t* test was applied. *p* = 0.05 was set as the threshold of significance.

## Results

### Baseline characterization of chimeras after bone marrow transplantation

#### Inflammation

6 weeks after bone marrow transplantation, blood counts of both groups did not differ except for mean platelet volume (BC+/EC+: 5.75 ± 0.33 μm^3^; *n* = 21 vs. BC−/EC+: 5.07 ± 0.18 μm^3^; *n* = 16 ***p < 0.001) and lymphocytes (BC+/EC+: 0.93 ± 0.23 10^3^/mm^3^; *n* = 21 vs. BC−/EC+: 1.69 ± 1.18 10^3^/mm^3^; *n* = 16; *p < 0.05) (See Online Resource 3 for detailed information). To analyze for chronic persisting inflammation as a result of the transplantation, serum amyloid P (SAP) levels were determined in plasma via ELISA. No differences were seen between the groups (BC+/EC+: 68.1 ± 6.2 μg/ml; *n* = 23 and BC−/EC+ 64.7 ± 9.7 μg/ml, *n* = 19; n.s.) in blood plasma 6 weeks after bone marrow transplantation (See Online Resource 4).

#### NO_x_ levels

**Fig. 1:**
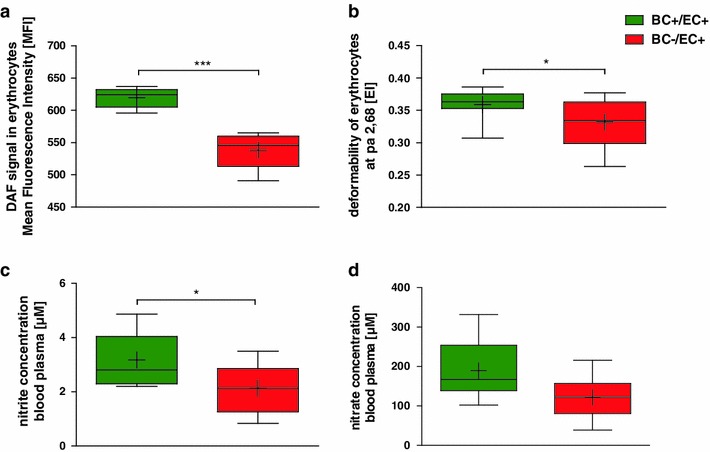
Depletion of circulating NOS3 reduces NO bioavailability, RBC deformability, and plasma levels of nitrite and nitrate. Decreased DAF signal within erythrocytes (**a**, *n* = 5 per group, ***p < 0.001, unpaired Student’s *t* test), reduced erythrocyte deformability (**b**, BC+/EC+ *n* = 15, BC−/EC+ *n* = 16, *p < 0.05, two-way ANOVA followed by Bonferroni’s post hoc test) and diminished nitrite (**c**; BC+/EC+ *n* = 10, BC−/EC+ *n* = 11, *p < 0.05, two-way ANOVA followed by Bonferroni’s post hoc test) and nitrate plasma levels (**d**; BC+/EC+ *n* = 10, BC−/EC+ *n* = 11; n.s.) were measured in BC−/EC+ compared to BC+/EC+ at baseline

BC−/EC+ chimera showed a decreased DAF-FM associated fluorescence within RBCs (BC−/EC+: 538.4 ± 12.8 MFI) as compared to BC+/EC+ mice (619.6 ± 6.9 MFI, ***p < 0.001, *n* = 5 per group) 6 weeks after bone marrow transplantation (See Fig. 1a). The background signal observed in RBC from BC−/EC+ mice is not different from that obtained by loading human RBC treated with L-NAME (observed before [5]), and is due to the formation of fluorescent adducts of DAF-FM with molecules such as ascorbate, particularly abundant in RBC, and to the presence of fluorescent impurities in the stock solutions of DAF-FM DA, which are detectable only by analytical separative techniques as described previously [4]. Results provided evidence for a diminished NO formation in RBC under normoxic conditions in BC−/EC+.

Nitrite levels in BC−/EC+ chimera (2.13 ± 0.27 μM; *n* = 11) were lower in plasma compared to BC+/EC+ mice (3.17 ± 0.29 μM; *n* = 10; *p < 0.05) at baseline (See Fig. 1c). Nitrate levels were slightly but not significantly reduced in BC−/EC+ (121.60 ± 16.37 μM; *n* = 11) compared to BC+/EC+ mice (189.09 ± 22.77 μM; *n* = 10; n.s.) at baseline (See Fig. 1d). Nitrite (BC+/EC+:1.36 ± 0.15 μM, *n* = 4 vs. BC−/EC+: 1.53 ± 0.22 μM, *n* = 3; n.s.) and nitrate (BC+/EC+:18.31 ± 4.10 μM, *n* = 4 vs. BC−/EC+: 27.07 ± 7.14 μM, *n* = 3; n.s.) levels in heart tissue did not differ between both groups at baseline (i.e., 6-week post-bone marrow transplantation) (See Online Resource 5). Likewise, nitrite (BC+/EC+: 1.07 ± 0.10 μM, *n* = 3 vs. BC−/EC+: 1.01 ± 0.07 μM, *n* = 3; n.s.) and nitrate (BC+/EC+: 41.61 ± 12.56 μM, *n* = 3 vs. BC−/EC+: 31.51 ± 6.81 μM, *n* = 3; n.s.) levels in aorta did not differ between both groups at baseline (i.e., 6-week post-bone marrow transplantation) (See Online Resource 6).

#### RBC deformability

6 weeks after bone marrow transplantation, BC−/EC+ (0.33 ± 0.01 EI; *n* = 16 per group) exhibited decreased RBC deformability compared to BC+/EC+ (0.36 ± 0.01 EI; *n* = 15; *p < 0.05) (See Fig. 1b). Both groups demonstrated diminished RBC deformability compared to non-irradiated wild type 6 weeks after transplantation (0.39 ± 0.01 EI; *n* = 15; ***p < 0.001).

#### Vascular reactivity

**Fig. 2:**
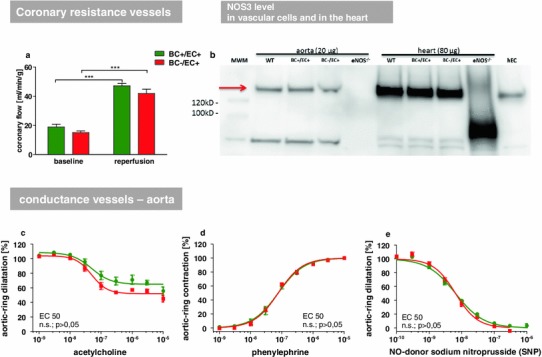
Comparison of NOS3 expression and endothelial function in chimeric and WT mice. Ex vivo measurements of coronary flow from isolated hearts in Langendorff setup (**a**) of BC+/EC+ and BC−/EC+ revealed no significant differences at baseline and after global ischemia (flow corrected for heart weight; BC+/EC+ *n* = 7, BC−/EC+ *n* = 8, n.s.). NOS3 expression in hearts from BC+/EC+, BC−/EC+ and WT mice was assessed by Western blot analysis and compared with NOS3 expression in aortic tissue. No differences were detected between the displayed groups, while heart tissue exhibited overall higher NOS3 level than aortic tissue (**b**). Isometric force measurements in aortic rings (**c**–**e**). BC+/EC+ and BC−/EC+ revealed no significant difference in the mean effective concentration (EC 50) and, therefore, no different vessel characteristics at baseline (*n* = 5 per group, n.s., EC50 tested with unpaired Student’s *t* test)

The effects of the transplantation procedure on vascular reactivity were assessed with three independent approaches. Ex vivo measurements of coronary flow (isolated hearts, Langendorff setup) of BC+/EC+ and BC−/EC+ revealed no differences in basal coronary flow (BC+/EC+: basal: 18.84 ± 1.91 ml/min/g vs. BC−/EC+: basal: 15.07 ± 1.08 ml/min/g, n.s.). After global brief ischemia, both groups responded with a uniform increase in coronary flow (BC+/EC+: basal: 18.84 ± 1.91 ml/min/g vs. reperfusion: 47.11 ± 1.67 ml/min/g, ***p < 0.001, *n* = 7; BC−/EC+: basal: 15.07 ± 1.08 ml/min/g vs. reperfusion: 41.90 ± 2.94 ml/min/g, ***p < 0.001, *n* = 8) (See Fig. 2a).

In vivo measurement of vascular reactivity was assessed by laser Doppler perfusion imaging, as changes in reactive hyperemic response following short-term vascular occlusion in the hind limb of BC+/EC+ and BC−/EC+ mice. The measurements revealed no differences in all parameters investigated (See Online Resource 7). Basal mean perfusion before induction of hind limb ischemia (BC+/EC+: 99 ± 5 %; *n* = 14; BC−/EC+: 106 ± 6 %; *n* = 12; n.s.), the time till reperfusion peak (maximum mean perfusion) (BC+/EC+: 42 ± 3 s; *n* = 14; BC−/EC+: 43 ± 3 s; *n* = 12; n.s.) and the area under the curve of the reperfusion signal (BC+/EC+: 1.106 ± 56; *n* = 14 BC−/EC+: 1.186 ± 91; *n* = 12; n.s.) did not differ between both groups.

Vascular reactivity of aortic rings from BC+/EC+ and BC−/EC+ mice was compared by wire myography. After pre-contraction with KCl response curves to acetylcholine, phenylephrine and SNP were determined as the response of EC50 to the applied substances. For all three conditions, no difference in the mean effective concentration (EC 50) and, therefore, no differences in endothelium-dependent and endothelium-independent vascular function were observed (See Fig. 2c–e).

#### NOS3 levels in heart and aorta

NOS3 expression in the heart and aorta from BC+/EC+, BC−/EC+ and WT mice was assessed by Western blot analysis. No differences were detected between the analyzed groups (See Fig. 2b).

#### Left ventricular function

Depletion of blood cell NOS3 did not modify left ventricular function at baseline (6 weeks after bone marrow transplantation) as determined by M-mode and B-mode measurements, indicating equal left ventricular function in BC−/EC+ compared to BC+/EC+.

Left ventricular ejection fraction (BC+/EC+: 61 ± 1 %; BC−/EC+: 62 ± 1 %; n.s), end-systolic volume (BC+/EC+: 25 ± 1 μl; BC−/EC+: 25 ± 1 μl; n.s) and end-diastolic volume (BC+/EC+: 66 ± 2 μl, *n* = 22; BC−/EC+: 66 ± 2 μl, *n* = 25; n.s.) did not differ between both groups (See Fig. 4 and Online Resource 8).

### Response of chimeras to myocardial ischemia/reperfusion

#### Blood cell NOS3 reduces infarct size following myocardial ischemia/reperfusion

**Fig. 3:**
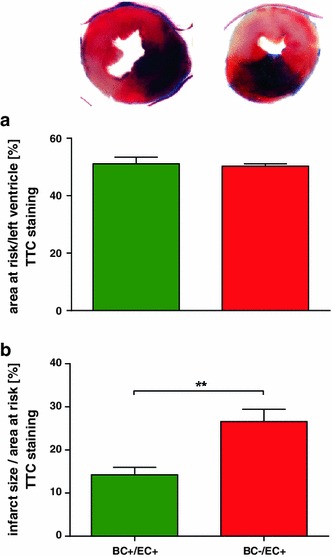
Depletion of circulating NOS3 increases infarct size following acute myocardial ischemia/reperfusion. While AAR per LV did not differ between both groups, infarct sizes were significantly increased in BC−/EC+ (*n* = 6) compared to BC+/EC+ (*n* = 9, **p < 0.01, unpaired Student’s *t* test)

Infarct size was increased in BC−/EC+ compared to BC+/EC+ (BC−/EC+: 26 ± 3 %; *n* = 6; BC+/EC+: 14 ± 2 %; *n* = 9 per group; **p < 0.01), while AAR per LV did not differ between both groups (BC−/EC+: 50 ± 1 %; *n* = 6 BC+/EC+: 51 ± 2 %; *n* = 9 per group; n.s.) (See Fig. 3).

#### Left ventricular function after 24 h of reperfusion

**Fig. 4:**
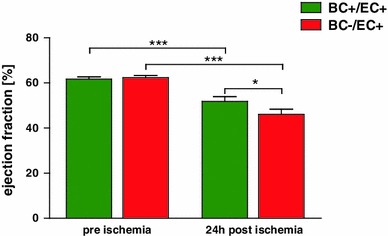
Depletion of circulating NOS3 impairs cardiac function following myocardial ischemia/reperfusion. After 60 min of ischemia and 24 h of reperfusion, ejection fraction (via echocardiography) was significantly impaired in BC+/EC+ (*n* = 21) and BC−/EC+ (*n* = 19) compared to baseline (BC+/EC+: *n* = 22, BC−/EC+: *n* = 25, ***p < 0.001 baseline vs. 24 h post ischemia, two-way ANOVA followed by Bonferroni’s post hoc test). Increased infarct size in BC−/EC+ was associated with significantly reduced ejection fraction compared to BC+/EC+ (*p < 0.05, two-way ANOVA followed by Bonferroni’s post hoc test)

After 24 h of reperfusion, systolic left ventricular function was impaired with reduced ejection fraction (BC−/EC+ 46 ± 2 %; *n* = 19 vs. BC+/EC+: 52 ± 2 %; *n* = 21; *p < 0.05) (See Fig. 4 and Online Resource 8) and increased end-systolic volume (BC−/EC+: 41 ± 2 μl; *n* = 19 vs. BC+/EC+: 34 ± 3 μl; *n* = 21; *p < 0.05) in BC−/EC+ compared to BC+/EC+ (See Online Resource 8).

#### ETU treatment

**Fig. 5:**
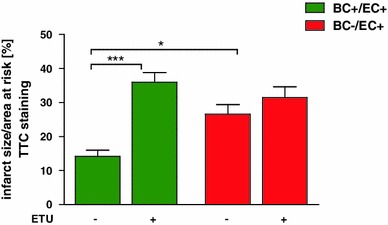
Circulating NOS3 contributes to reduced infarct size. Application of the global NOS inhibitor S-ethylisothiourea hydrobromide (ETU) was associated with a further increase in infarct size in BC+/EC+ (without ETU *n* = 9; with ETU *n* = 6; ***p < 0.001; one-way ANOVA followed by Bonferroni’s post hoc test), whereas BC−/EC+ (without and with ETU *n* = 6) demonstrated no significant differences after global NOS inhibition

Application of the global NOS inhibitor ETU was associated with increased infarct size (ETU: 36 ± 3 %; *n* = 6 vs. without ETU: 14 ± 2 %; *n* = 9; *p < 0.05) in BC+/EC+, whereas BC−/EC+ demonstrated no differences after global NOS inhibition (ETU: 32 ± 3 %; *n* = 6 without ETU: 27 ± 3 %; *n* = 6; n.s.) (See Fig. 5).

#### Nitrite in early and late reperfusion: blood cell NOS3 contributes to endogenous NO pool and improves NO bioavailability in the early reperfusion phase

Blood plasma nitrite levels were slightly reduced in BC−/EC+ plasma at 5 min (BC−/EC+: 0.90 ± 0.18 μM vs. BC+/EC+:1.95 ± 0.41 μM; *n* = 4 per group; n.s.) and 24 h of reperfusion (BC−/EC+: 1.21 ± 0.18 μM; *n* = 7 vs. BC+/EC+: 1.56 ± 0.26 μM; *n* = 6; n.s.) compared to BC+/EC+ (See Online Resource 9a). Nitrate concentration in plasma was significantly reduced in BC−/EC+ (5 min: 95.45 ± 21.40 μM; *n* = 4 and 24 h: 74.38 ± 7.42 μM; *n* = 5) compared to BC+/EC+ (5 min: 224.12 ± 57.12 μM; *n* = 4; *p < 0.05 and 24 h: 178.84 ± 16.37 μM; *n* = 6; *p < 0.05) at 5 min and 24 h of reperfusion (See Online Resource 9b). Nitrite and nitrate concentration in heart tissue did not differ between both groups at 5 min (nitrite: BC+/EC+: 1.42 ± 0.24 μM; BC−/EC+: 1.36 ± 0.23 μM, *n* = 4 per group, n.s.; nitrate: BC+/EC+: 21.92 ± 1.74 μM, *n* = 3 vs. BC−/EC+: 12.32 ± 2.17 μM, *n* = 4, n.s) and 24 h of reperfusion (nitrite: BC+/EC+: 0.41 ± 0.06 μM vs. BC−/EC+: 0.31 ± 0.12 μM, *n* = 3 per group, n.s.; nitrate: BC+/EC+: 12.78 ± 0.48 μM vs. BC−/EC+: 13.67 ± 4.09 μM, *n* = 3 per group; n.s.).

#### Blood cell NOS3 preserved erythrocyte deformability 24-h post-ischemia

RBC deformability was diminished in BC−/EC+ (0.27 ± 0.01 EI; *n* = 16) compared to BC+/EC+ (0.35 ± 0.01 EI; *n* = 15; ***p < 0.001) 24 h after reperfusion. Furthermore, BC−/EC+ showed a decrease in RBC deformability after 24 h of reperfusion compared to baseline (0.33 ± 0.01 EI; *n* = 16; **p < 0.05). In contrast, RBC deformability was not affected by I/R in BC+/EC+ chimeras (0.36 ± 0.06 EI; *n* = 15; n.s.) (See Online Resource 9c).

## Discussion

The main findings of the present study are: (1) circulating NOS3 reduces infarct size in a myocardial ischemia/reperfusion model of chimeric mice; (2) this translates into a sustained reduction of LV function; (3) depletion of circulating NOS3 reduces circulating NO pool by 1/3, as evidenced by reduced plasma levels of nitrite; (4) circulating NOS3 limits RBC deformability.

In the present study, depletion of circulating NOS3 is associated with increased infarct size and impaired left ventricular function. In accordance with these findings, global depletion of NOS3^−/−^ increased infarct size and impaired LV function after I/R [20], and conventional wisdom holds that these effects are primarily determined by the lack of NOS3 in the vessel endothelium. Our data suggest that endothelial NOS3 is unable to compensate for the depletion of circulating NOS3 alone in terms of reduction of tissue damage and preservation of left ventricular function as a result of myocardial I/R in the current chimeric model. It is conceivable that endothelial dysfunction, which occurs during I/R and is associated with endothelial NOS3 uncoupling, limits the functional capacity of endothelial NOS3 [8]. We did not find any differences in vascular function in the two mice groups, as assessed in both conductance and resistance vessels. Thus, we exclude that differences in endothelial functionality between groups might have caused differences in infarct size. In the context of myocardial ischemia, NOS inhibition exacerbates myocardial I/R injury [25]. In our study, application of the global NOS inhibitor ETU was associated with increased infarct size in BC+/EC+, whereas BC−/EC+ demonstrated no differences after global NOS inhibition. Therefore, circulating NOS3 limits infarct size and this translates into preserved LV function in a myocardial I/R model.

We examined nitrite and nitrate levels in plasma and heart tissue as a marker of systemic NO bioavailability at baseline, 5 min and 24 h after ischemia. Both groups showed a significant decrease in nitrite plasma levels 5 min after ischemia compared to baseline. Whereas mice with depleted circulating NOS3 show more reduced plasma nitrite levels compared to animals carrying circulating NOS at baseline and 5 min after ischemia. However, nitrite heart tissue levels remain stable and show no difference between both groups at all examined time points. Both groups are obviously able to maintain their nitrite/nitrate concentration to a certain degree in the analyzed tissues (heart and aorta) at baseline and at least in the first 5 min of reperfusion independently of surrounding plasma levels. Therefore, depletion of circulating NOS3 was associated with nitrite levels reduced by 1/3 at baseline compared to animals carrying circulating NOS3 only in plasma but not in heart tissue. These data confirm and expand recent findings in chimera obtained from the two further existing NOS3^−/−^ strains [47] other than the one used in this study, and demonstrate that circulating NOS3 plays a central role in determining the circulating NO pool. These reduced levels of circulating nitrite likely contribute to the greater tissue damage in BC−/EC+ chimeras, as previously shown in WT mice [13, 14]. Nevertheless, the exact quantitative contribution of circulating NOS3 to the circulating nitric oxide metabolite (NO_x_) pool cannot be assessed accurately without analyzing nitrite/nitrate content in whole blood and the differentially in the individual blood cell types as well as plasma. While this was not primarily the aim of the present study, it certainly represents a limitation and should be addressed in further studies.

The organism is able to reduce nitrite to NO, thus potentially compensating for an impaired NOS activity/NO bioavailability, [6]. Nitrite is, therefore, considered as an “ischemic NO buffer” [14]. Various nitrite reductases exist, which are effective at different oxygen partial pressures along the vascular tree: (1) hemoglobin, which reduces nitrite at 20–60 mmHg oxygen, (2) myoglobin, which is active below 4 mmHg oxygen, and (3) xanthine oxidase and the acidic reduction which reduce nitrite at 0 mmHg oxygen and low pH [28]. In mice feed with a controlled nitrite/nitrate low diet, both the circulating and the endothelial NOS3 contribute to 70–90 % of the total plasma nitrite levels [22, 24]. Chimeras exhibit elevated plasma nitrite levels compared to non-transplanted wild types. This might be related to a chronic inflammation within the bone marrow due to a subclinical rejection [27]. A strong persisting systemic inflammation was excluded in the chimeras of the present study by determining the concentration of SAP in the blood plasma, a well-established murine marker of inflammation [37], and by serial assessment of white blood cells counts. As discussed above, the lower nitrite levels in BC−/EC+ chimera indicate the contribution of circulating NOS3 to nitrite bioavailability. NO itself and nitrite, both emerging from constitutive endothelial and blood born NOS3 activity, may contribute to the cardioprotective effect in the reperfused myocardium.

During circulation, RBCs constantly change their shape as they are exposed to a range of dynamic shear stresses, and are able to respond to these forces by changing their shape [3]. This property of RBCs is defined as deformability and contributes to blood fluidity, blood flow and the passage of RBCs through capillaries with diameters smaller than their resting diameter [35, 49]. In the present study, we found that depletion of circulating NOS3 was associated with reduced RBC deformability, as previously shown in human RBC treated with NOS inhibitors [2] and a further reduction of RBC deformability was measured in BC−/EC+ mice following myocardial I/R and may contribute to myocardial damage. In fact, in patients with coronary artery disease (CAD) and diabetes mellitus, pathological alterations of hemostatic and hemorheological properties have been found to be associated with an increased incidence of coronary events [18, 19] and contribute to increased morbidity of these patients due to disturbances in blood flow [21]. Furthermore, it is known that oxidative stress and impaired NO bioavailability are able to reduce the deformability of RBCs [34]. A more extensive reduction of NO bioavailability and increase of ROS concentration within RBCs are probable reasons for the further decrease in deformability of RBCs in the BC−/EC+ group compared to group BC+/EC+ after myocardial I/R. The preserved deformability of RBCs as observed in BC+/EC+ mice might positively impact coronary blood flow particularly in the border zone of an ischemic area and thus might also contribute to reduce infarct size and limited left ventricular function in our model of myocardial I/R.

In conclusion, we here present evidence that circulating NOS3 reduced infarct size leading to sustained left ventricular dysfunction in an acute model of myocardial I/R in chimeric mice. Depletion of circulating NOS3 reduces NO bioavailability and limits RBC deformability in this model. For future analyses of mechanisms underlying the protective effects of circulating NOS3 in I/R injury, it would certainly be beneficial to use a conditional erythrocyte-specific knockout model to exclude any confounding factors due to irradiation/bone marrow transplantation and to identify the specific role of blood cell subpopulations in these effects.

## Electronic supplementary material

Below is the link to the electronic supplementary material.

Supplementary material 1 (PPTX 60 kb)

Supplementary material 2 (PPTX 67 kb)

Supplementary material 3 (PPTX 62 kb)

Supplementary material 4 (PPTX 62 kb)

Supplementary material 5 (PPTX 97 kb)

Supplementary material 6 (PPTX 72 kb)

Supplementary material 7 (PPTX 70 kb)

Supplementary material 8 (PPTX 63 kb)

Supplementary material 9 (PPTX 66 kb)
