# Supplementary material for: Depletion of circulating blood NOS3 increases severity of myocardial infarction and left ventricular dysfunction
Source: Basic Res Cardiol. 2013 Dec 18;109(1):398. doi: 10.1007/s00395-013-0398-1 (PMC3898535; doi:10.1007/s00395-013-0398-1)
Supplement: Supplementary file 1 — Supplementary material 1 (PPTX 60 kb) [file 395_2013_398_MOESM1_ESM.pptx]

## Slide 1
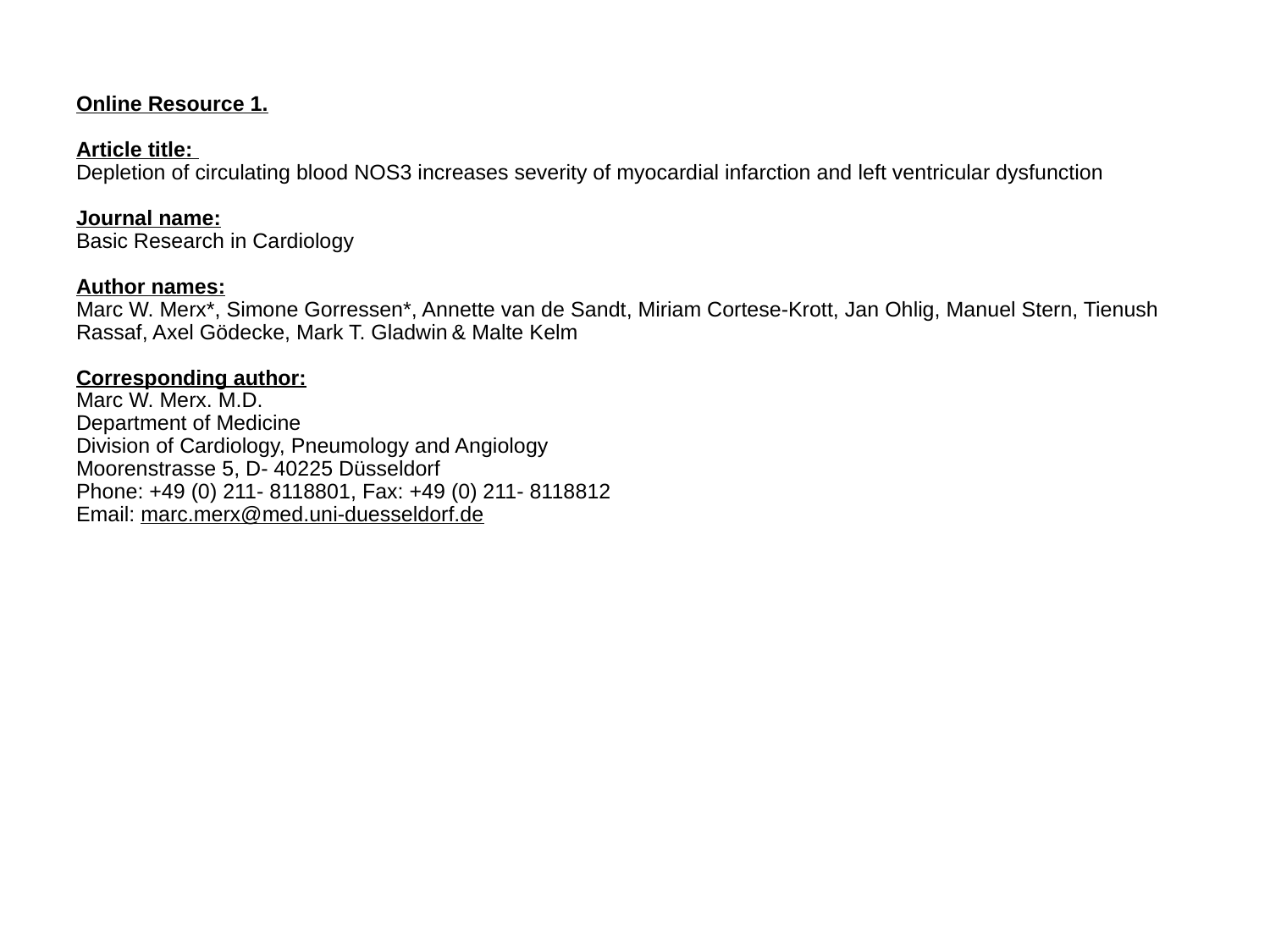

# Online Resource 1. Article title: Depletion of circulating blood NOS3 increases severity of myocardial infarction and left ventricular dysfunction Journal name: Basic Research in Cardiology Author names: Marc W. Merx*, Simone Gorressen*, Annette van de Sandt, Miriam Cortese-Krott, Jan Ohlig, Manuel Stern, Tienush Rassaf, Axel Gödecke, Mark T. Gladwin & Malte Kelm Corresponding author: Marc W. Merx. M.D. Department of Medicine Division of Cardiology, Pneumology and Angiology Moorenstrasse 5, D- 40225 Düsseldorf Phone: +49 (0) 211- 8118801, Fax: +49 (0) 211- 8118812 Email: marc.merx@med.uni-duesseldorf.de

## Slide 2
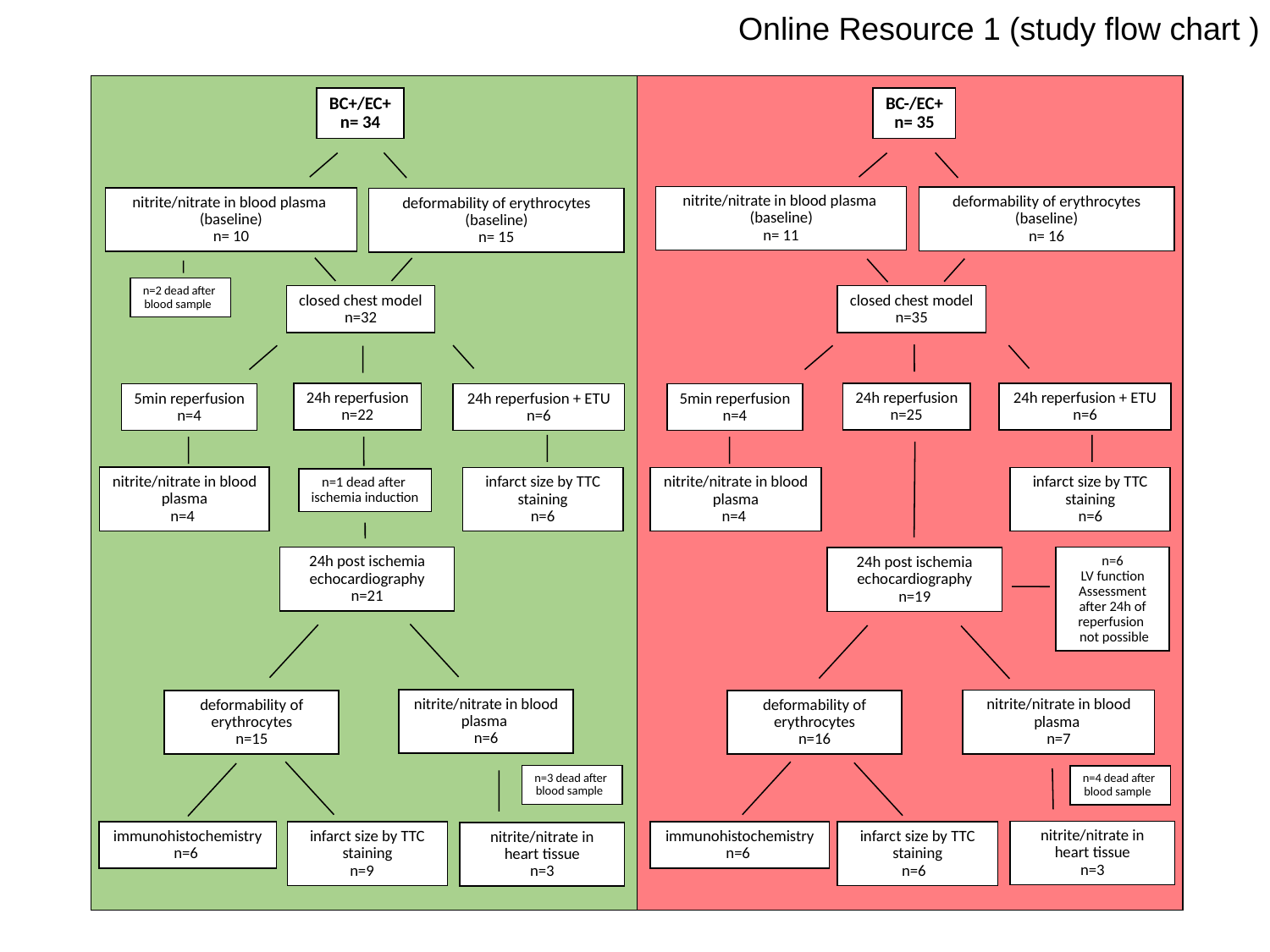

Online Resource 1 (study flow chart )
BC+/EC+
n= 34
BC-/EC+
n= 35
nitrite/nitrate in blood plasma
(baseline)
n= 11
deformability of erythrocytes (baseline)
n= 16
nitrite/nitrate in blood plasma
(baseline)
n= 10
deformability of erythrocytes (baseline)
n= 15
n=2 dead after
blood sample
closed chest model
n=32
closed chest model
n=35
24h reperfusion
n=22
24h reperfusion
n=25
24h reperfusion + ETU
n=6
5min reperfusion
n=4
24h reperfusion + ETU
n=6
5min reperfusion
n=4
nitrite/nitrate in blood plasma
n=4
infarct size by TTC staining
n=6
nitrite/nitrate in blood plasma
n=4
infarct size by TTC staining
n=6
n=1 dead after
ischemia induction
24h post ischemia echocardiography
n=21
n=6
LV function
Assessment
after 24h of reperfusion
 not possible
24h post ischemia echocardiography
n=19
nitrite/nitrate in blood plasma
n=6
nitrite/nitrate in blood plasma
n=7
deformability of erythrocytes
n=15
deformability of erythrocytes
n=16
n=3 dead after
blood sample
n=4 dead after
blood sample
nitrite/nitrate in heart tissue
n=3
immunohistochemistry
n=6
infarct size by TTC staining
n=9
immunohistochemistry
n=6
infarct size by TTC staining
n=6
nitrite/nitrate in heart tissue
n=3

## Slide 3
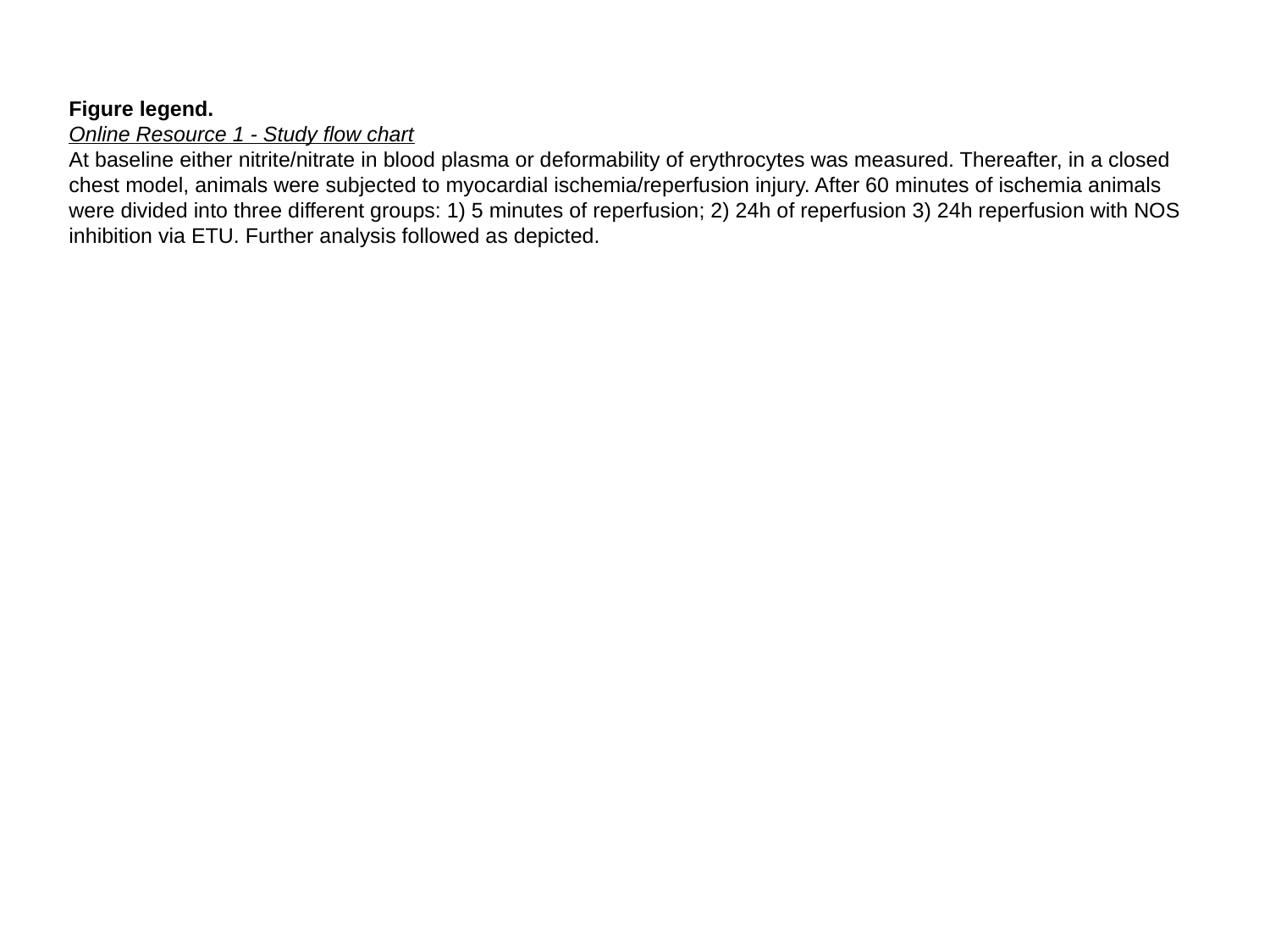

Figure legend.
Online Resource 1 - Study flow chart
At baseline either nitrite/nitrate in blood plasma or deformability of erythrocytes was measured. Thereafter, in a closed chest model, animals were subjected to myocardial ischemia/reperfusion injury. After 60 minutes of ischemia animals were divided into three different groups: 1) 5 minutes of reperfusion; 2) 24h of reperfusion 3) 24h reperfusion with NOS inhibition via ETU. Further analysis followed as depicted.
