# Supplementary material for: Depletion of circulating blood NOS3 increases severity of myocardial infarction and left ventricular dysfunction
Source: Basic Res Cardiol. 2013 Dec 18;109(1):398. doi: 10.1007/s00395-013-0398-1 (PMC3898535; doi:10.1007/s00395-013-0398-1)
Supplement: Supplementary file 2 — Supplementary material 2 (PPTX 67 kb) [file 395_2013_398_MOESM2_ESM.pptx]

## Slide 1
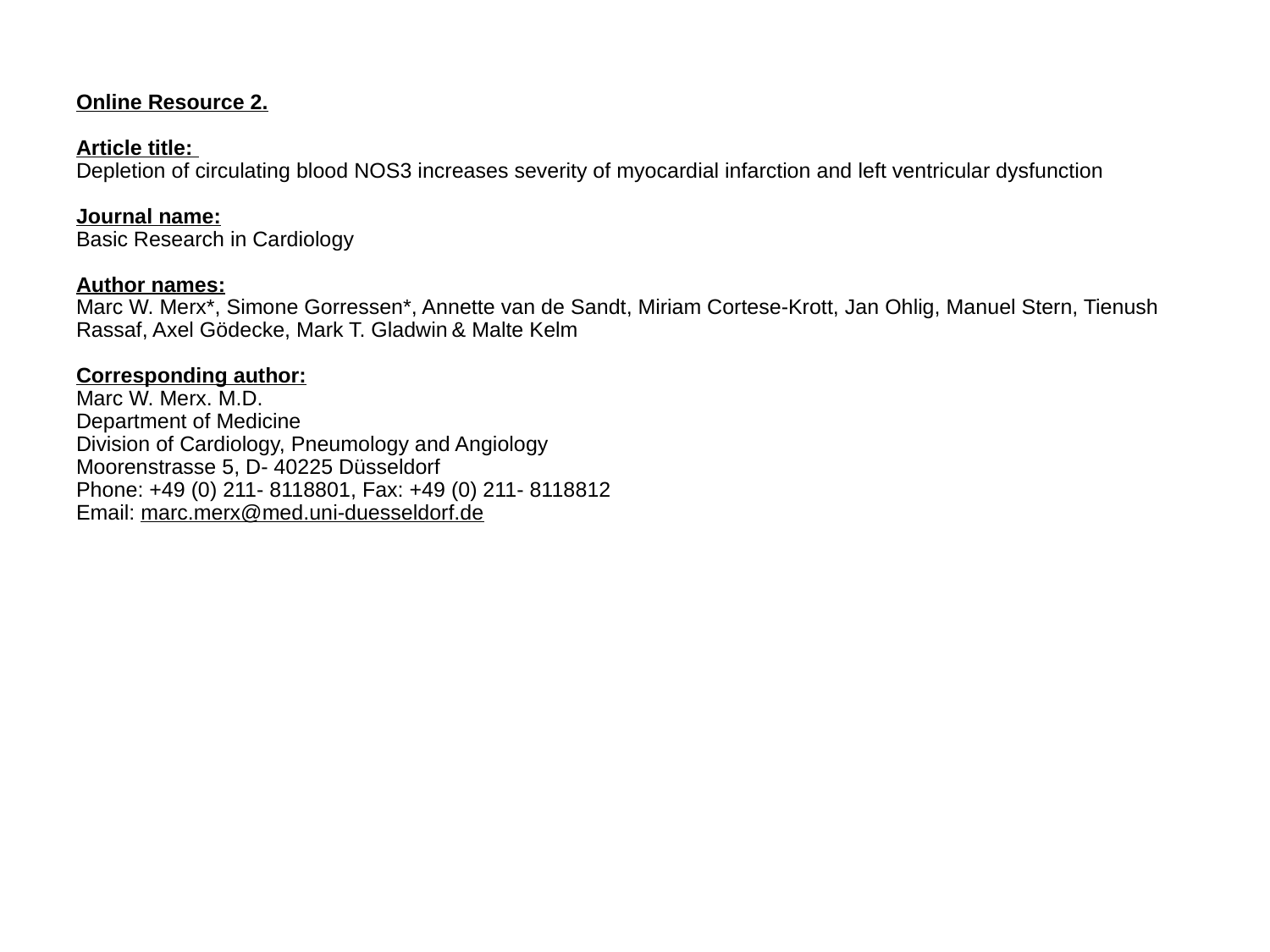

# Online Resource 2. Article title: Depletion of circulating blood NOS3 increases severity of myocardial infarction and left ventricular dysfunction Journal name: Basic Research in Cardiology Author names: Marc W. Merx*, Simone Gorressen*, Annette van de Sandt, Miriam Cortese-Krott, Jan Ohlig, Manuel Stern, Tienush Rassaf, Axel Gödecke, Mark T. Gladwin & Malte Kelm Corresponding author: Marc W. Merx. M.D. Department of Medicine Division of Cardiology, Pneumology and Angiology Moorenstrasse 5, D- 40225 Düsseldorf Phone: +49 (0) 211- 8118801, Fax: +49 (0) 211- 8118812 Email: marc.merx@med.uni-duesseldorf.de

## Slide 2
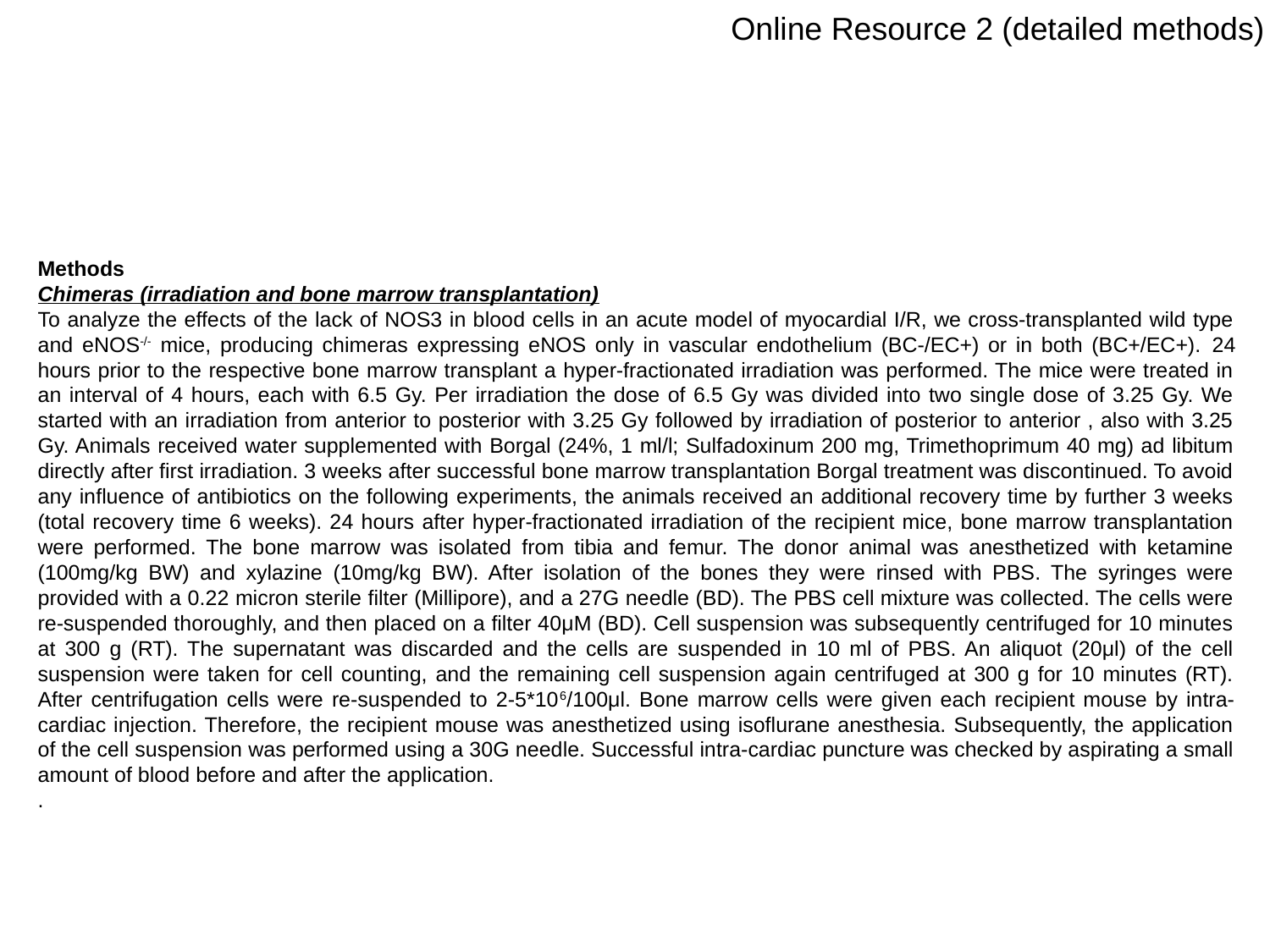

Online Resource 2 (detailed methods)
Methods
Chimeras (irradiation and bone marrow transplantation)
To analyze the effects of the lack of NOS3 in blood cells in an acute model of myocardial I/R, we cross-transplanted wild type and eNOS-/- mice, producing chimeras expressing eNOS only in vascular endothelium (BC-/EC+) or in both (BC+/EC+). 24 hours prior to the respective bone marrow transplant a hyper-fractionated irradiation was performed. The mice were treated in an interval of 4 hours, each with 6.5 Gy. Per irradiation the dose of 6.5 Gy was divided into two single dose of 3.25 Gy. We started with an irradiation from anterior to posterior with 3.25 Gy followed by irradiation of posterior to anterior , also with 3.25 Gy. Animals received water supplemented with Borgal (24%, 1 ml/l; Sulfadoxinum 200 mg, Trimethoprimum 40 mg) ad libitum directly after first irradiation. 3 weeks after successful bone marrow transplantation Borgal treatment was discontinued. To avoid any influence of antibiotics on the following experiments, the animals received an additional recovery time by further 3 weeks (total recovery time 6 weeks). 24 hours after hyper-fractionated irradiation of the recipient mice, bone marrow transplantation were performed. The bone marrow was isolated from tibia and femur. The donor animal was anesthetized with ketamine (100mg/kg BW) and xylazine (10mg/kg BW). After isolation of the bones they were rinsed with PBS. The syringes were provided with a 0.22 micron sterile filter (Millipore), and a 27G needle (BD). The PBS cell mixture was collected. The cells were re-suspended thoroughly, and then placed on a filter 40μM (BD). Cell suspension was subsequently centrifuged for 10 minutes at 300 g (RT). The supernatant was discarded and the cells are suspended in 10 ml of PBS. An aliquot (20μl) of the cell suspension were taken for cell counting, and the remaining cell suspension again centrifuged at 300 g for 10 minutes (RT). After centrifugation cells were re-suspended to 2-5*106/100μl. Bone marrow cells were given each recipient mouse by intra-cardiac injection. Therefore, the recipient mouse was anesthetized using isoflurane anesthesia. Subsequently, the application of the cell suspension was performed using a 30G needle. Successful intra-cardiac puncture was checked by aspirating a small amount of blood before and after the application.
.

## Slide 3
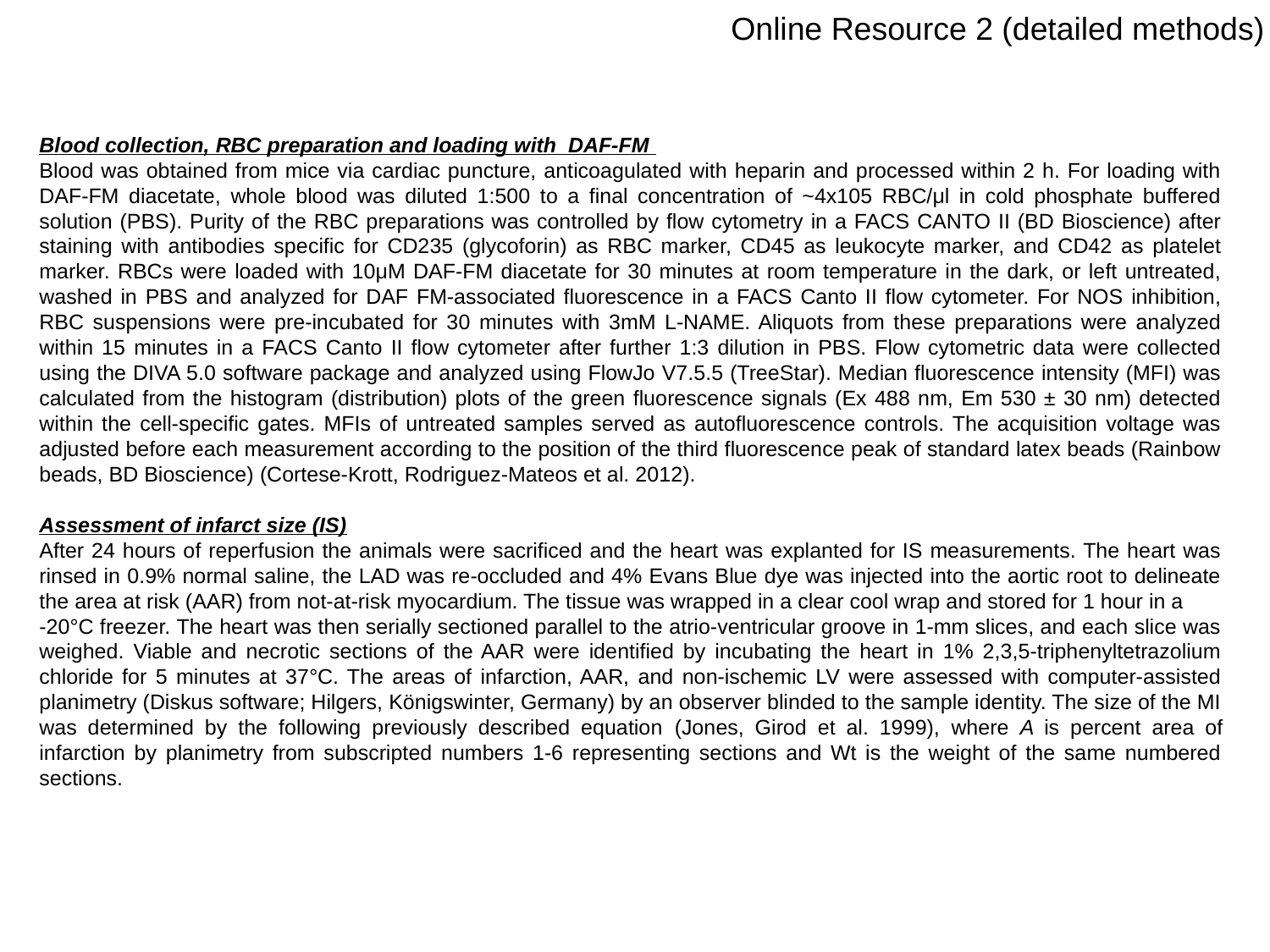

Online Resource 2 (detailed methods)
Blood collection, RBC preparation and loading with DAF-FM
Blood was obtained from mice via cardiac puncture, anticoagulated with heparin and processed within 2 h. For loading with DAF-FM diacetate, whole blood was diluted 1:500 to a final concentration of ~4x10^5^ RBC/μl in cold phosphate buffered solution (PBS). Purity of the RBC preparations was controlled by flow cytometry in a FACS CANTO II (BD Bioscience) after staining with antibodies specific for CD235 (glycoforin) as RBC marker, CD45 as leukocyte marker, and CD42 as platelet marker. RBCs were loaded with 10μM DAF-FM diacetate for 30 minutes at room temperature in the dark, or left untreated, washed in PBS and analyzed for DAF FM-associated fluorescence in a FACS Canto II flow cytometer. For NOS inhibition, RBC suspensions were pre-incubated for 30 minutes with 3mM L-NAME. Aliquots from these preparations were analyzed within 15 minutes in a FACS Canto II flow cytometer after further 1:3 dilution in PBS. Flow cytometric data were collected using the DIVA 5.0 software package and analyzed using FlowJo V7.5.5 (TreeStar). Median fluorescence intensity (MFI) was calculated from the histogram (distribution) plots of the green fluorescence signals (Ex 488 nm, Em 530 ± 30 nm) detected within the cell-specific gates. MFIs of untreated samples served as autofluorescence controls. The acquisition voltage was adjusted before each measurement according to the position of the third fluorescence peak of standard latex beads (Rainbow beads, BD Bioscience) (Cortese-Krott, Rodriguez-Mateos et al. 2012).
Assessment of infarct size (IS)
After 24 hours of reperfusion the animals were sacrificed and the heart was explanted for IS measurements. The heart was rinsed in 0.9% normal saline, the LAD was re-occluded and 4% Evans Blue dye was injected into the aortic root to delineate the area at risk (AAR) from not-at-risk myocardium. The tissue was wrapped in a clear cool wrap and stored for 1 hour in a
-20°C freezer. The heart was then serially sectioned parallel to the atrio-ventricular groove in 1-mm slices, and each slice was weighed. Viable and necrotic sections of the AAR were identified by incubating the heart in 1% 2,3,5-triphenyltetrazolium chloride for 5 minutes at 37°C. The areas of infarction, AAR, and non-ischemic LV were assessed with computer-assisted planimetry (Diskus software; Hilgers, Königswinter, Germany) by an observer blinded to the sample identity. The size of the MI was determined by the following previously described equation (Jones, Girod et al. 1999), where A is percent area of infarction by planimetry from subscripted numbers 1-6 representing sections and Wt is the weight of the same numbered sections.

## Slide 4
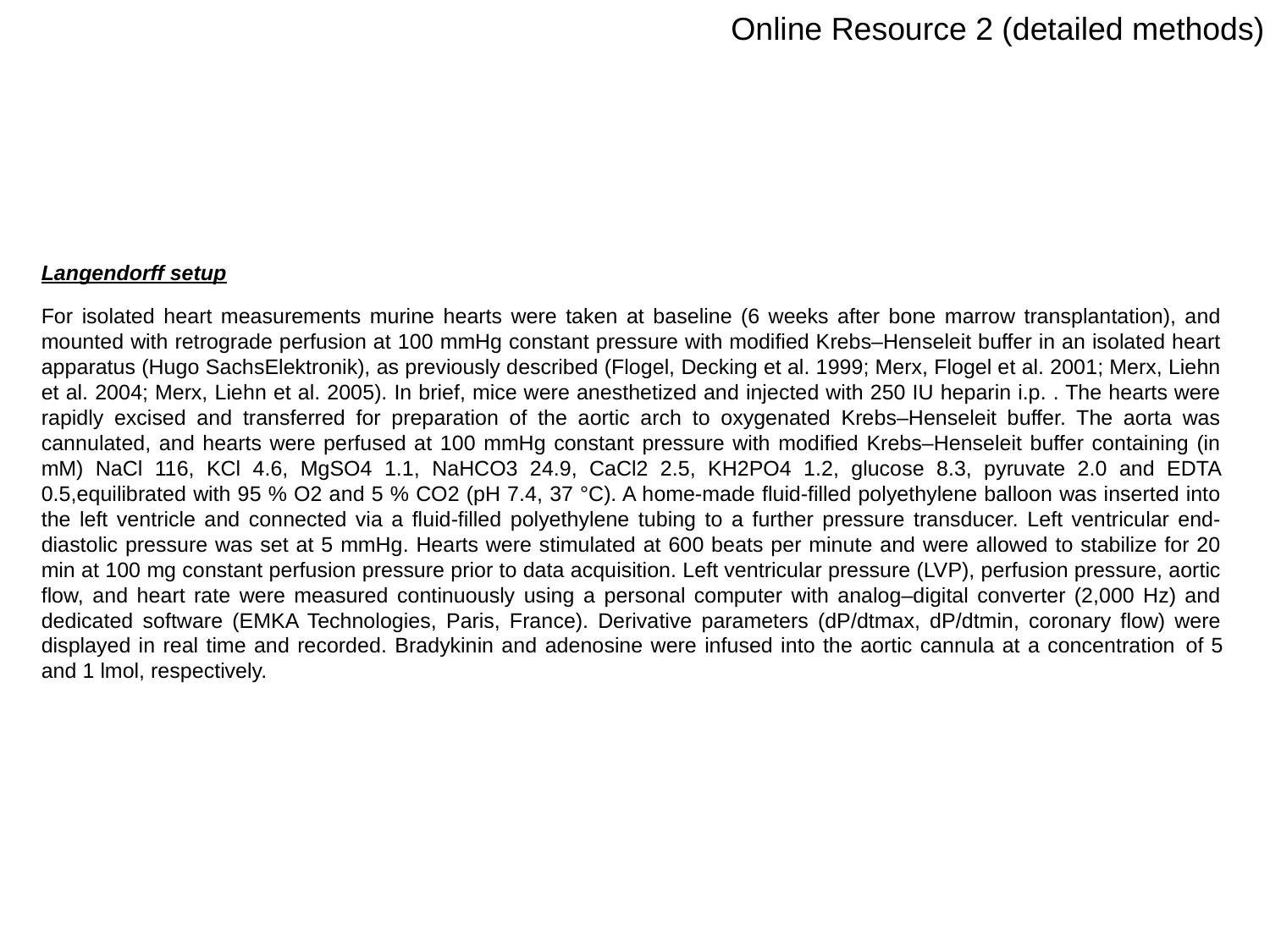

Online Resource 2 (detailed methods)
Langendorff setup
For isolated heart measurements murine hearts were taken at baseline (6 weeks after bone marrow transplantation), and mounted with retrograde perfusion at 100 mmHg constant pressure with modified Krebs–Henseleit buffer in an isolated heart apparatus (Hugo SachsElektronik), as previously described (Flogel, Decking et al. 1999; Merx, Flogel et al. 2001; Merx, Liehn et al. 2004; Merx, Liehn et al. 2005). In brief, mice were anesthetized and injected with 250 IU heparin i.p. . The hearts were rapidly excised and transferred for preparation of the aortic arch to oxygenated Krebs–Henseleit buffer. The aorta was cannulated, and hearts were perfused at 100 mmHg constant pressure with modified Krebs–Henseleit buffer containing (in mM) NaCl 116, KCl 4.6, MgSO4 1.1, NaHCO3 24.9, CaCl2 2.5, KH2PO4 1.2, glucose 8.3, pyruvate 2.0 and EDTA 0.5,equilibrated with 95 % O2 and 5 % CO2 (pH 7.4, 37 °C). A home-made fluid-filled polyethylene balloon was inserted into the left ventricle and connected via a fluid-filled polyethylene tubing to a further pressure transducer. Left ventricular end-diastolic pressure was set at 5 mmHg. Hearts were stimulated at 600 beats per minute and were allowed to stabilize for 20 min at 100 mg constant perfusion pressure prior to data acquisition. Left ventricular pressure (LVP), perfusion pressure, aortic flow, and heart rate were measured continuously using a personal computer with analog–digital converter (2,000 Hz) and dedicated software (EMKA Technologies, Paris, France). Derivative parameters (dP/dtmax, dP/dtmin, coronary flow) were displayed in real time and recorded. Bradykinin and adenosine were infused into the aortic cannula at a concentration of 5 and 1 lmol, respectively.

## Slide 5
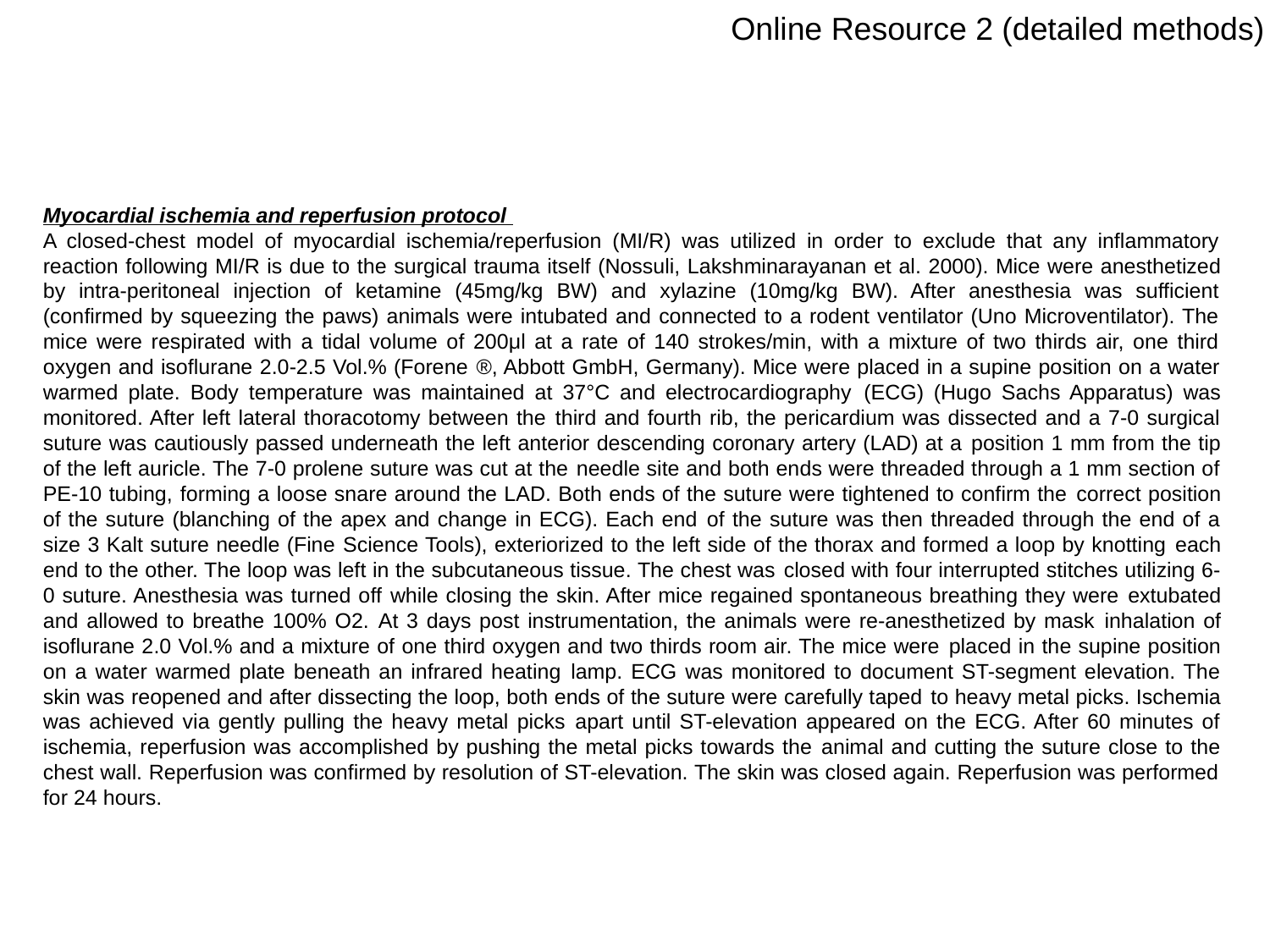

Online Resource 2 (detailed methods)
Myocardial ischemia and reperfusion protocol
A closed-chest model of myocardial ischemia/reperfusion (MI/R) was utilized in order to exclude that any inflammatory reaction following MI/R is due to the surgical trauma itself (Nossuli, Lakshminarayanan et al. 2000). Mice were anesthetized by intra-peritoneal injection of ketamine (45mg/kg BW) and xylazine (10mg/kg BW). After anesthesia was sufficient (confirmed by squeezing the paws) animals were intubated and connected to a rodent ventilator (Uno Microventilator). The mice were respirated with a tidal volume of 200μl at a rate of 140 strokes/min, with a mixture of two thirds air, one third oxygen and isoflurane 2.0-2.5 Vol.% (Forene ®, Abbott GmbH, Germany). Mice were placed in a supine position on a water warmed plate. Body temperature was maintained at 37°C and electrocardiography (ECG) (Hugo Sachs Apparatus) was monitored. After left lateral thoracotomy between the third and fourth rib, the pericardium was dissected and a 7-0 surgical suture was cautiously passed underneath the left anterior descending coronary artery (LAD) at a position 1 mm from the tip of the left auricle. The 7-0 prolene suture was cut at the needle site and both ends were threaded through a 1 mm section of PE-10 tubing, forming a loose snare around the LAD. Both ends of the suture were tightened to confirm the correct position of the suture (blanching of the apex and change in ECG). Each end of the suture was then threaded through the end of a size 3 Kalt suture needle (Fine Science Tools), exteriorized to the left side of the thorax and formed a loop by knotting each end to the other. The loop was left in the subcutaneous tissue. The chest was closed with four interrupted stitches utilizing 6-0 suture. Anesthesia was turned off while closing the skin. After mice regained spontaneous breathing they were extubated and allowed to breathe 100% O2. At 3 days post instrumentation, the animals were re-anesthetized by mask inhalation of isoflurane 2.0 Vol.% and a mixture of one third oxygen and two thirds room air. The mice were placed in the supine position on a water warmed plate beneath an infrared heating lamp. ECG was monitored to document ST-segment elevation. The skin was reopened and after dissecting the loop, both ends of the suture were carefully taped to heavy metal picks. Ischemia was achieved via gently pulling the heavy metal picks apart until ST-elevation appeared on the ECG. After 60 minutes of ischemia, reperfusion was accomplished by pushing the metal picks towards the animal and cutting the suture close to the chest wall. Reperfusion was confirmed by resolution of ST-elevation. The skin was closed again. Reperfusion was performed for 24 hours.
