# Supplementary material for: Depletion of circulating blood NOS3 increases severity of myocardial infarction and left ventricular dysfunction
Source: Basic Res Cardiol. 2013 Dec 18;109(1):398. doi: 10.1007/s00395-013-0398-1 (PMC3898535; doi:10.1007/s00395-013-0398-1)
Supplement: Supplementary file 3 — Supplementary material 3 (PPTX 62 kb) [file 395_2013_398_MOESM3_ESM.pptx]

## Slide 1
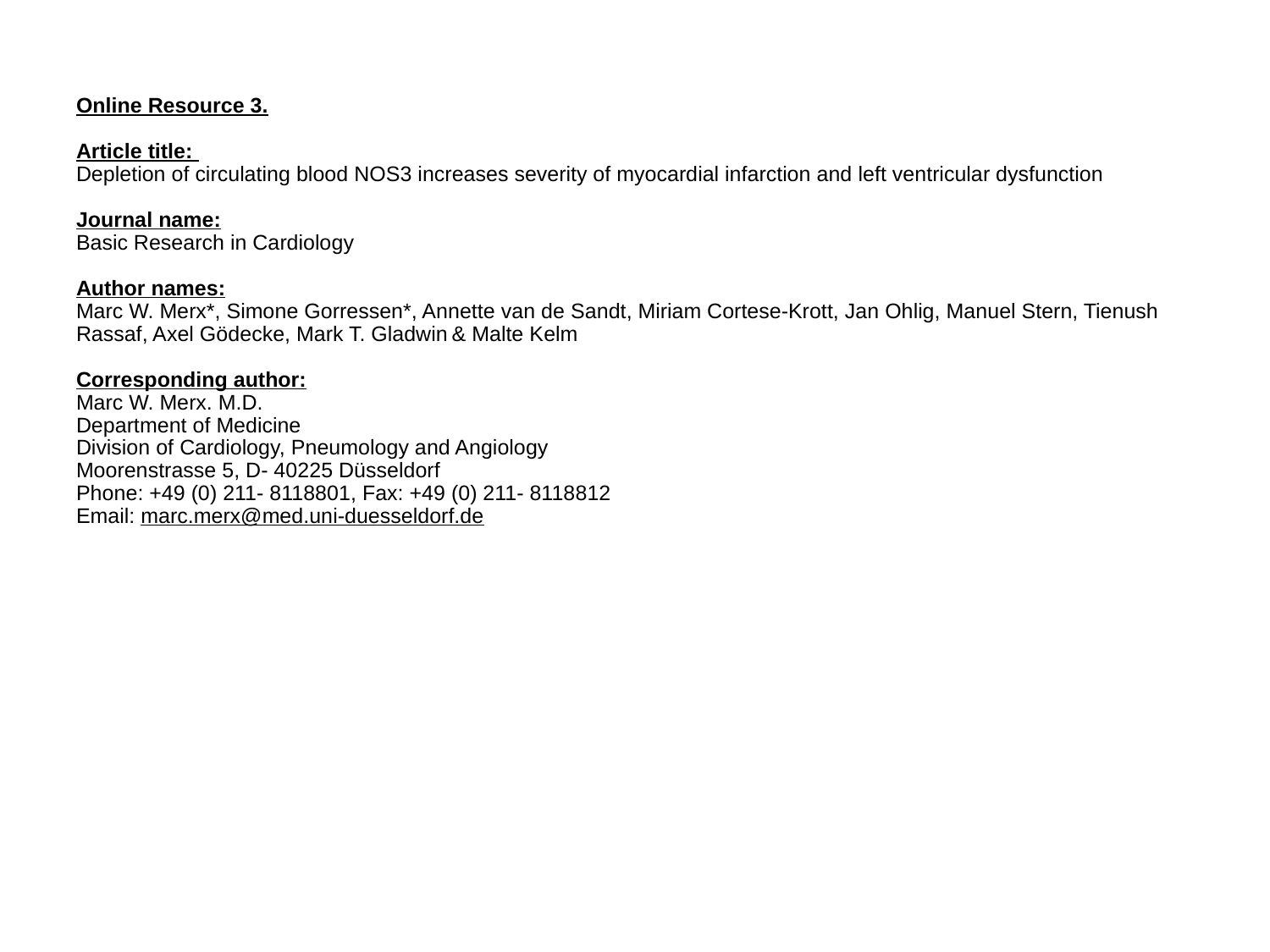

# Online Resource 3. Article title: Depletion of circulating blood NOS3 increases severity of myocardial infarction and left ventricular dysfunction Journal name: Basic Research in Cardiology Author names: Marc W. Merx*, Simone Gorressen*, Annette van de Sandt, Miriam Cortese-Krott, Jan Ohlig, Manuel Stern, Tienush Rassaf, Axel Gödecke, Mark T. Gladwin & Malte Kelm Corresponding author: Marc W. Merx. M.D. Department of Medicine Division of Cardiology, Pneumology and Angiology Moorenstrasse 5, D- 40225 Düsseldorf Phone: +49 (0) 211- 8118801, Fax: +49 (0) 211- 8118812 Email: marc.merx@med.uni-duesseldorf.de

## Slide 2
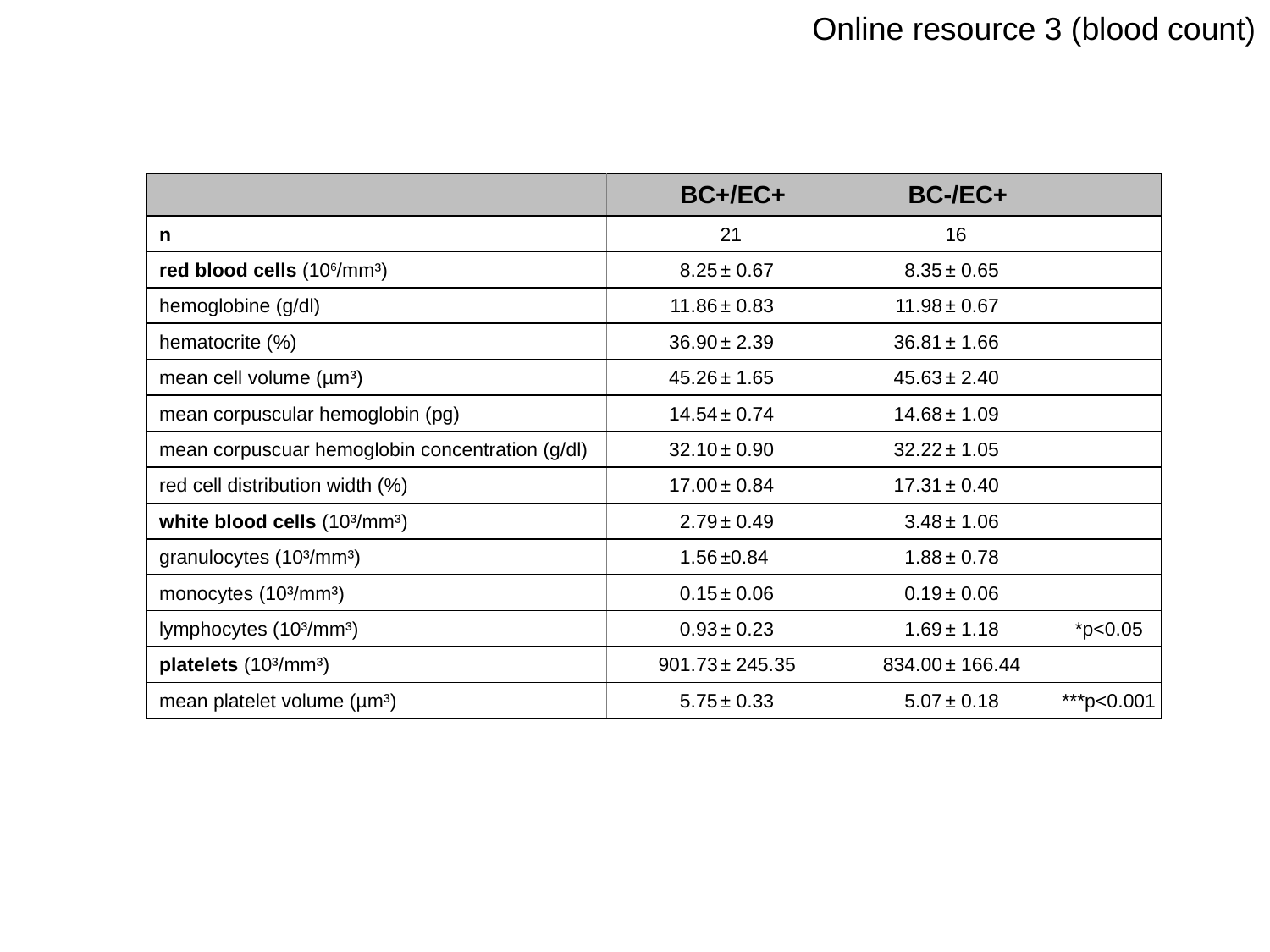

Online resource 3 (blood count)
| | BC+/EC+ | | BC-/EC+ | | |
| --- | --- | --- | --- | --- | --- |
| n | | 21 | | 16 | |
| red blood cells (106/mm³) | 8.25 | ± 0.67 | 8.35 | ± 0.65 | |
| hemoglobine (g/dl) | 11.86 | ± 0.83 | 11.98 | ± 0.67 | |
| hematocrite (%) | 36.90 | ± 2.39 | 36.81 | ± 1.66 | |
| mean cell volume (µm³) | 45.26 | ± 1.65 | 45.63 | ± 2.40 | |
| mean corpuscular hemoglobin (pg) | 14.54 | ± 0.74 | 14.68 | ± 1.09 | |
| mean corpuscuar hemoglobin concentration (g/dl) | 32.10 | ± 0.90 | 32.22 | ± 1.05 | |
| red cell distribution width (%) | 17.00 | ± 0.84 | 17.31 | ± 0.40 | |
| white blood cells (10³/mm³) | 2.79 | ± 0.49 | 3.48 | ± 1.06 | |
| granulocytes (10³/mm³) | 1.56 | ±0.84 | 1.88 | ± 0.78 | |
| monocytes (10³/mm³) | 0.15 | ± 0.06 | 0.19 | ± 0.06 | |
| lymphocytes (10³/mm³) | 0.93 | ± 0.23 | 1.69 | ± 1.18 | \*p<0.05 |
| platelets (10³/mm³) | 901.73 | ± 245.35 | 834.00 | ± 166.44 | |
| mean platelet volume (µm³) | 5.75 | ± 0.33 | 5.07 | ± 0.18 | \*\*\*p<0.001 |

## Slide 3
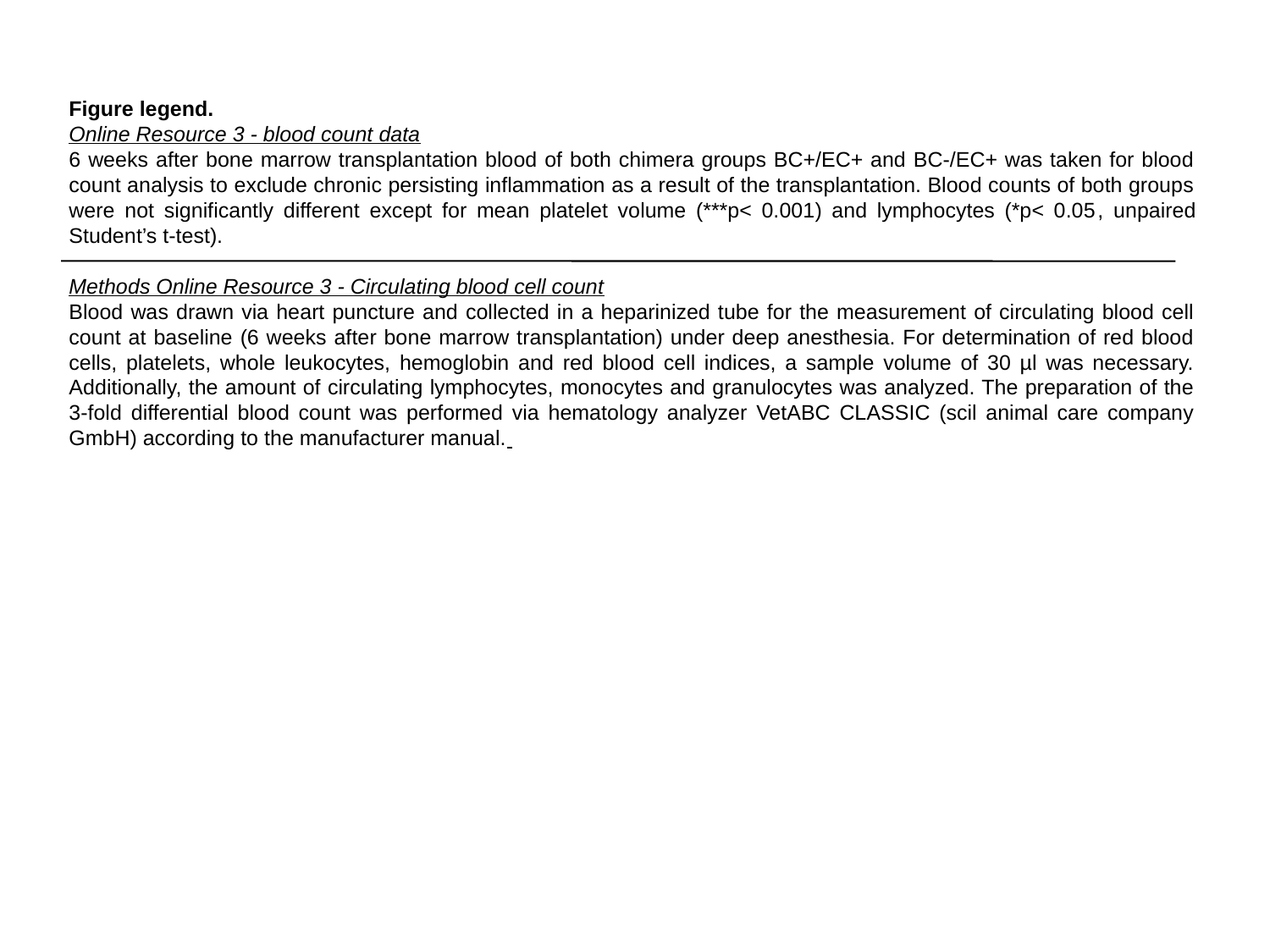

Figure legend.
Online Resource 3 - blood count data
6 weeks after bone marrow transplantation blood of both chimera groups BC+/EC+ and BC-/EC+ was taken for blood count analysis to exclude chronic persisting inflammation as a result of the transplantation. Blood counts of both groups were not significantly different except for mean platelet volume (***p< 0.001) and lymphocytes (*p< 0.05, unpaired Student’s t-test).
Methods Online Resource 3 - Circulating blood cell count
Blood was drawn via heart puncture and collected in a heparinized tube for the measurement of circulating blood cell count at baseline (6 weeks after bone marrow transplantation) under deep anesthesia. For determination of red blood cells, platelets, whole leukocytes, hemoglobin and red blood cell indices, a sample volume of 30 µl was necessary. Additionally, the amount of circulating lymphocytes, monocytes and granulocytes was analyzed. The preparation of the 3-fold differential blood count was performed via hematology analyzer VetABC CLASSIC (scil animal care company GmbH) according to the manufacturer manual.
