# Supplementary material for: Depletion of circulating blood NOS3 increases severity of myocardial infarction and left ventricular dysfunction
Source: Basic Res Cardiol. 2013 Dec 18;109(1):398. doi: 10.1007/s00395-013-0398-1 (PMC3898535; doi:10.1007/s00395-013-0398-1)
Supplement: Supplementary file 4 — Supplementary material 4 (PPTX 62 kb) [file 395_2013_398_MOESM4_ESM.pptx]

## Slide 1
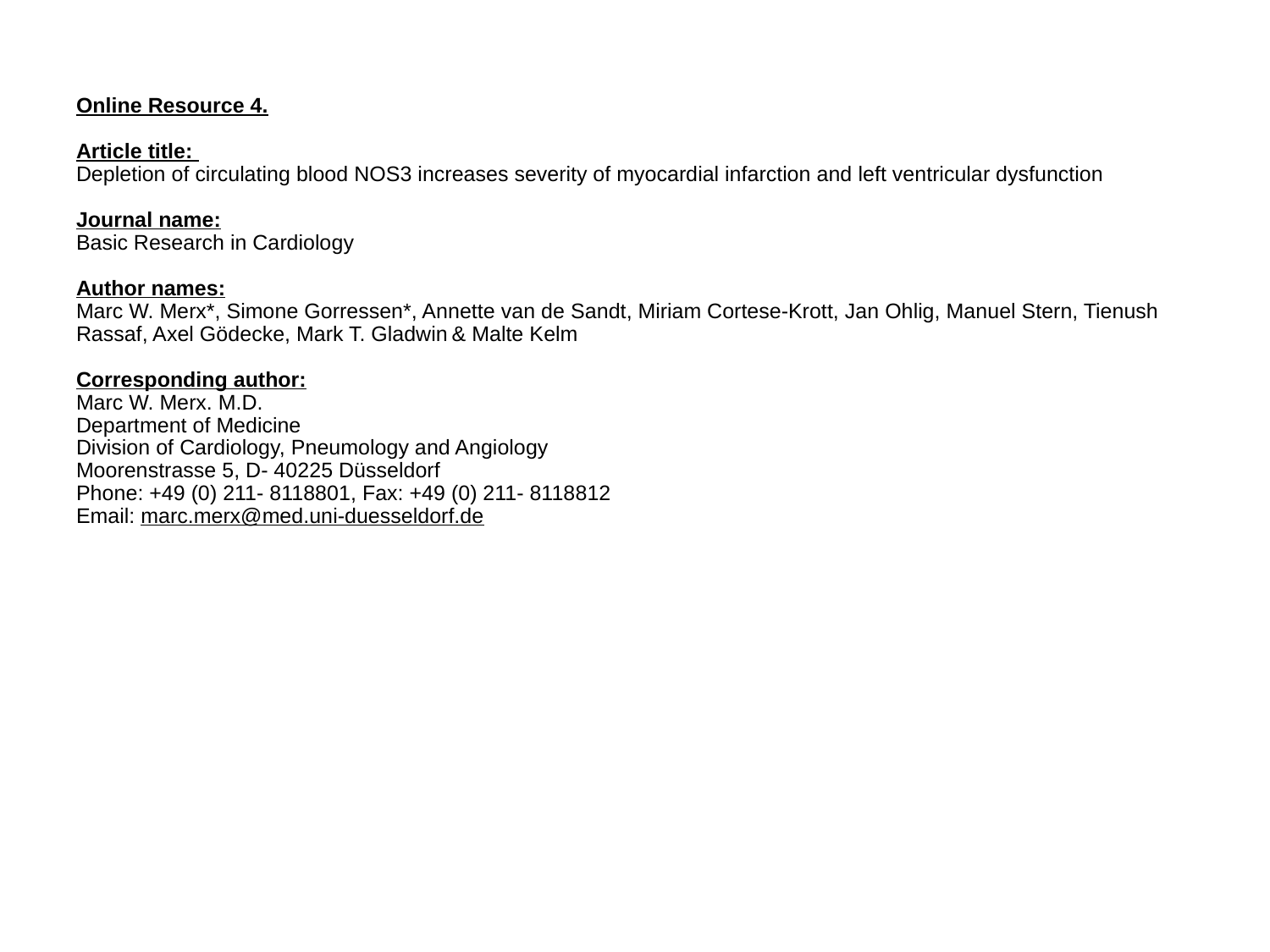

# Online Resource 4. Article title: Depletion of circulating blood NOS3 increases severity of myocardial infarction and left ventricular dysfunction Journal name: Basic Research in Cardiology Author names: Marc W. Merx*, Simone Gorressen*, Annette van de Sandt, Miriam Cortese-Krott, Jan Ohlig, Manuel Stern, Tienush Rassaf, Axel Gödecke, Mark T. Gladwin & Malte Kelm Corresponding author: Marc W. Merx. M.D. Department of Medicine Division of Cardiology, Pneumology and Angiology Moorenstrasse 5, D- 40225 Düsseldorf Phone: +49 (0) 211- 8118801, Fax: +49 (0) 211- 8118812 Email: marc.merx@med.uni-duesseldorf.de

## Slide 2
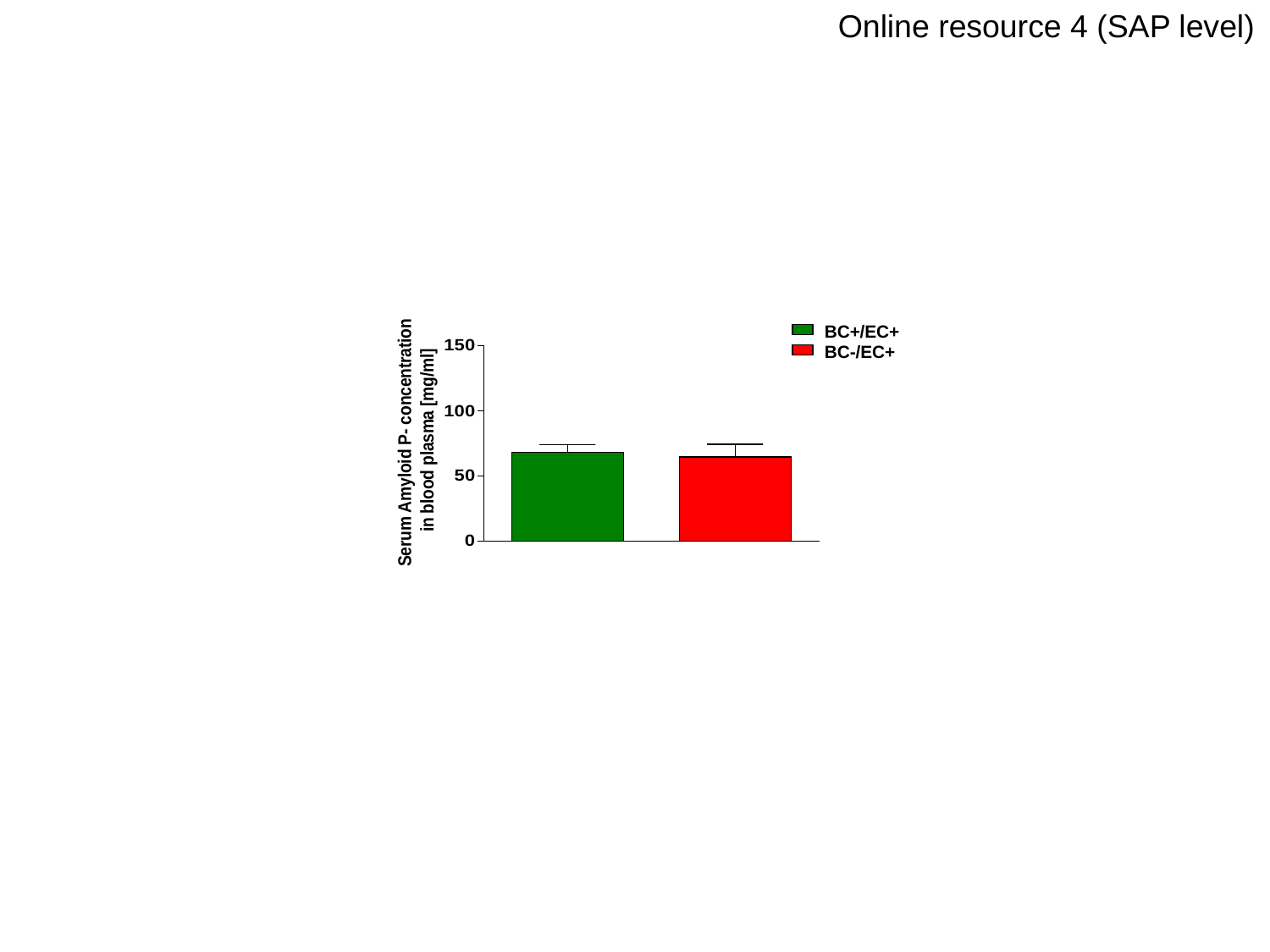

Online resource 4 (SAP level)
BC+/EC+
BC-/EC+

## Slide 3
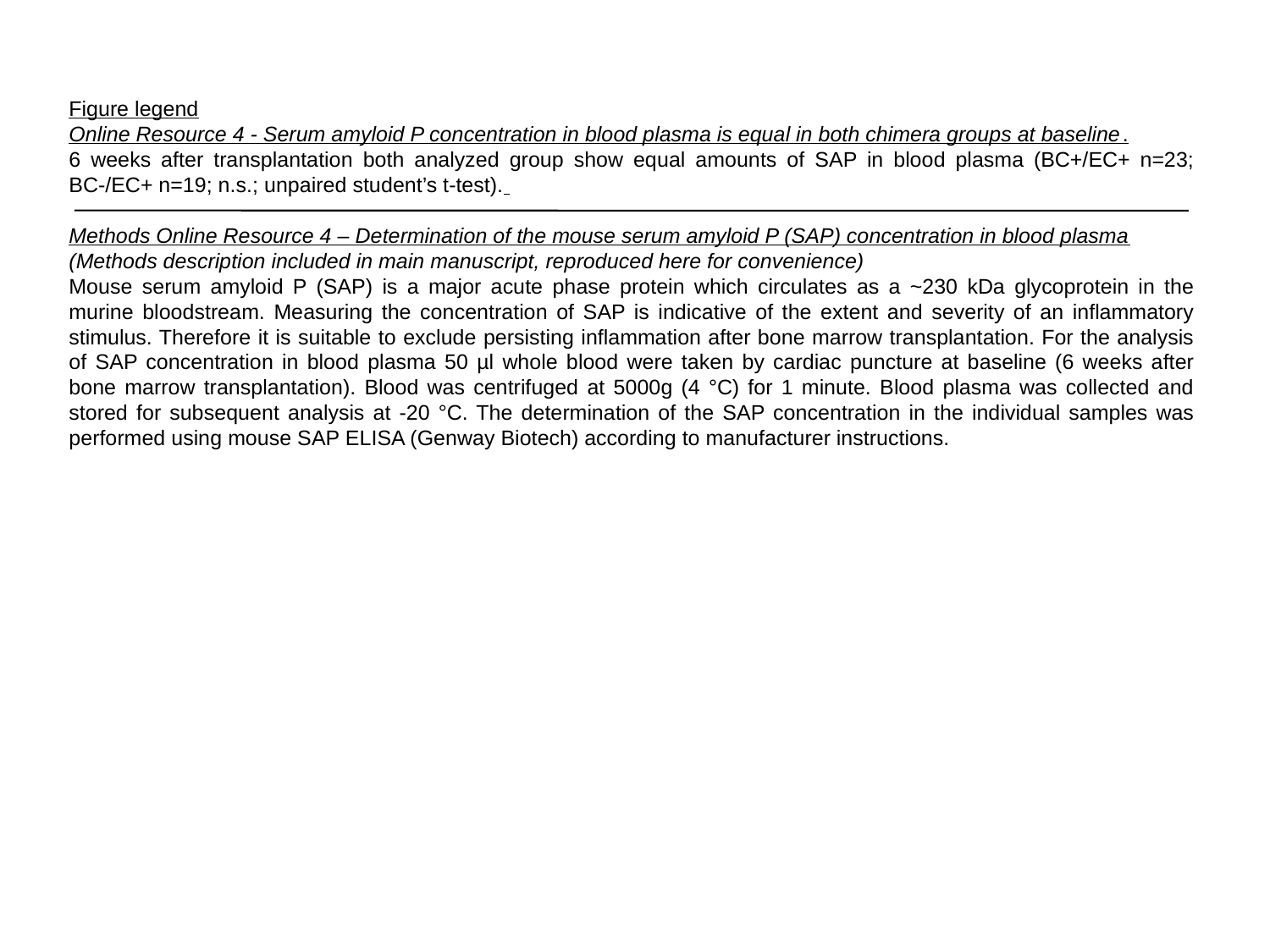

Figure legend
Online Resource 4 - Serum amyloid P concentration in blood plasma is equal in both chimera groups at baseline.
6 weeks after transplantation both analyzed group show equal amounts of SAP in blood plasma (BC+/EC+ n=23; BC-/EC+ n=19; n.s.; unpaired student’s t-test).
Methods Online Resource 4 – Determination of the mouse serum amyloid P (SAP) concentration in blood plasma
(Methods description included in main manuscript, reproduced here for convenience)
Mouse serum amyloid P (SAP) is a major acute phase protein which circulates as a ~230 kDa glycoprotein in the murine bloodstream. Measuring the concentration of SAP is indicative of the extent and severity of an inflammatory stimulus. Therefore it is suitable to exclude persisting inflammation after bone marrow transplantation. For the analysis of SAP concentration in blood plasma 50 µl whole blood were taken by cardiac puncture at baseline (6 weeks after bone marrow transplantation). Blood was centrifuged at 5000g (4 °C) for 1 minute. Blood plasma was collected and stored for subsequent analysis at -20 °C. The determination of the SAP concentration in the individual samples was performed using mouse SAP ELISA (Genway Biotech) according to manufacturer instructions.
