# Supplementary material for: Depletion of circulating blood NOS3 increases severity of myocardial infarction and left ventricular dysfunction
Source: Basic Res Cardiol. 2013 Dec 18;109(1):398. doi: 10.1007/s00395-013-0398-1 (PMC3898535; doi:10.1007/s00395-013-0398-1)
Supplement: Supplementary file 5 — Supplementary material 5 (PPTX 97 kb) [file 395_2013_398_MOESM5_ESM.pptx]

## Slide 1
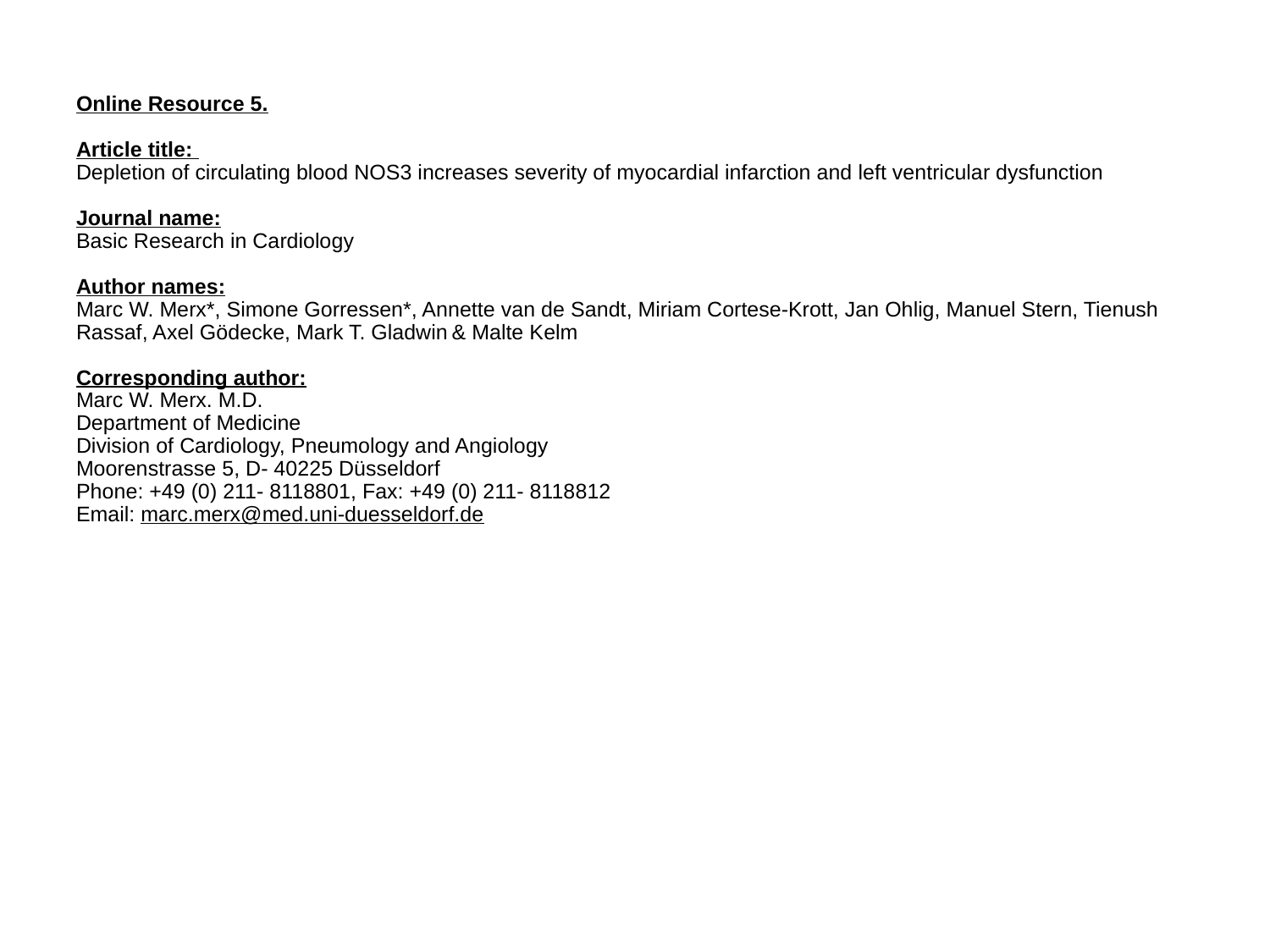

# Online Resource 5. Article title: Depletion of circulating blood NOS3 increases severity of myocardial infarction and left ventricular dysfunction Journal name: Basic Research in Cardiology Author names: Marc W. Merx*, Simone Gorressen*, Annette van de Sandt, Miriam Cortese-Krott, Jan Ohlig, Manuel Stern, Tienush Rassaf, Axel Gödecke, Mark T. Gladwin & Malte Kelm Corresponding author: Marc W. Merx. M.D. Department of Medicine Division of Cardiology, Pneumology and Angiology Moorenstrasse 5, D- 40225 Düsseldorf Phone: +49 (0) 211- 8118801, Fax: +49 (0) 211- 8118812 Email: marc.merx@med.uni-duesseldorf.de

## Slide 2
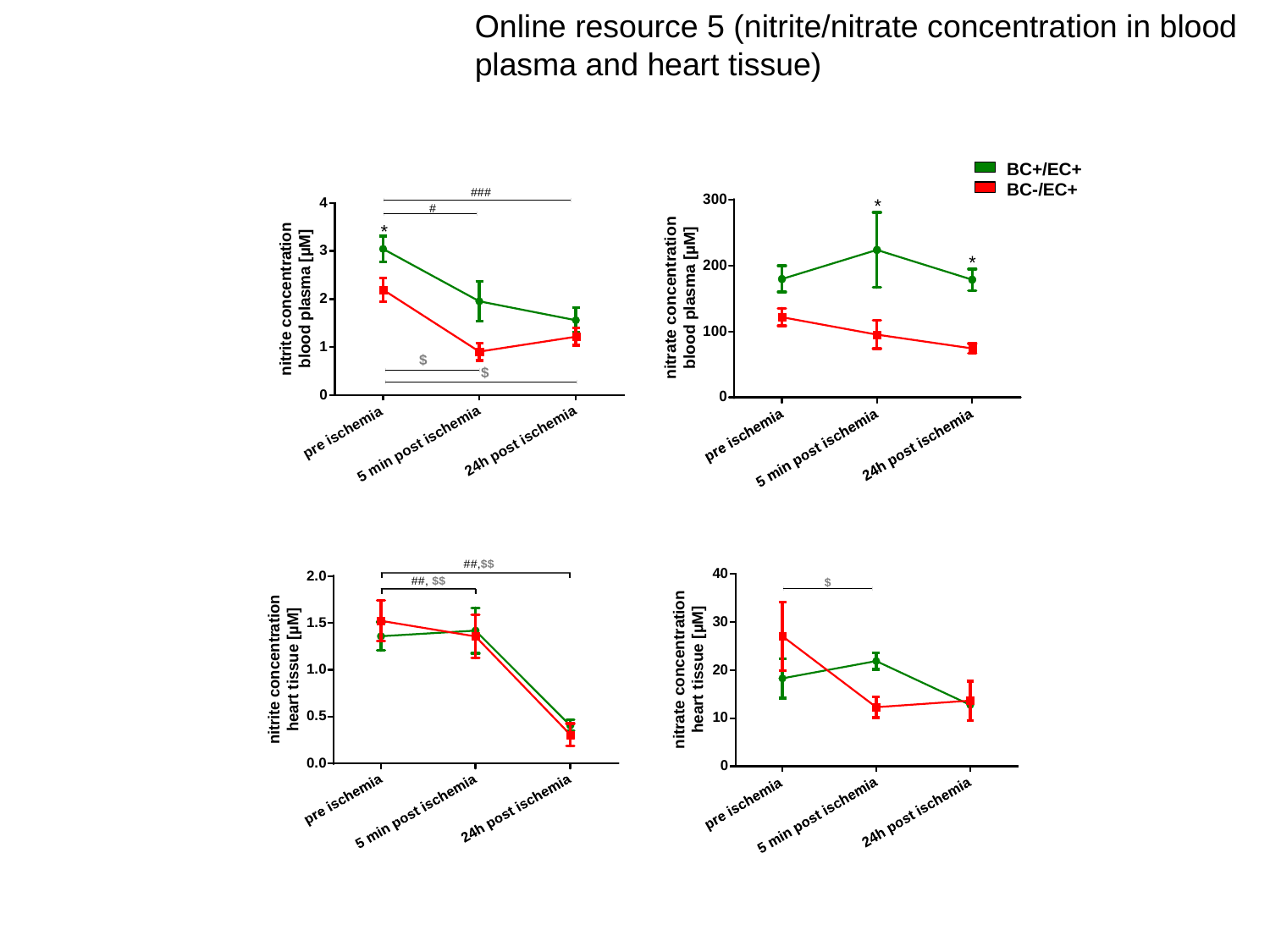

Online resource 5 (nitrite/nitrate concentration in blood
plasma and heart tissue)
BC+/EC+
BC-/EC+

## Slide 3
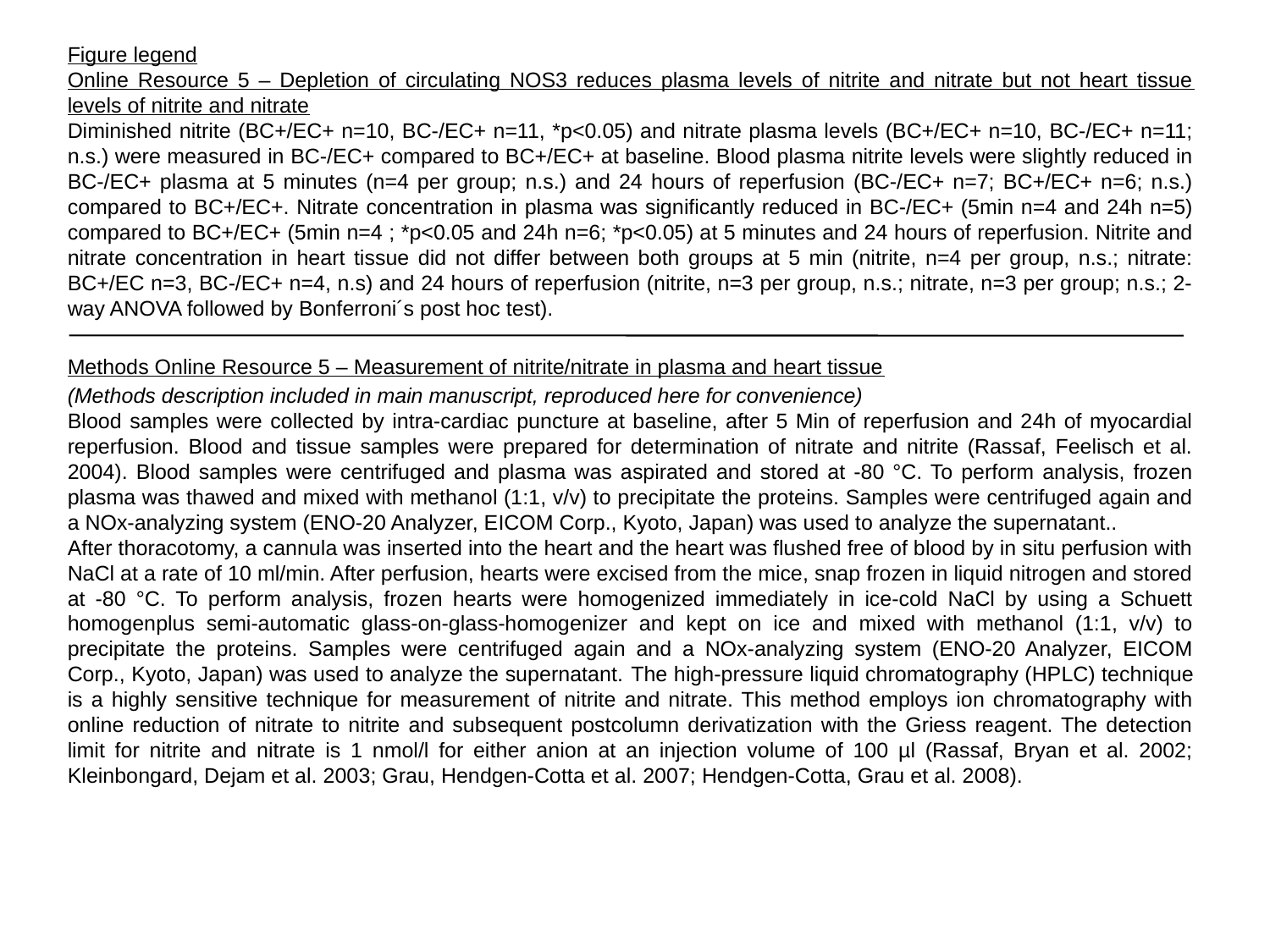

Figure legend
Online Resource 5 – Depletion of circulating NOS3 reduces plasma levels of nitrite and nitrate but not heart tissue levels of nitrite and nitrate
Diminished nitrite (BC+/EC+ n=10, BC-/EC+ n=11, *p<0.05) and nitrate plasma levels (BC+/EC+ n=10, BC-/EC+ n=11; n.s.) were measured in BC-/EC+ compared to BC+/EC+ at baseline. Blood plasma nitrite levels were slightly reduced in BC-/EC+ plasma at 5 minutes (n=4 per group; n.s.) and 24 hours of reperfusion (BC-/EC+ n=7; BC+/EC+ n=6; n.s.) compared to BC+/EC+. Nitrate concentration in plasma was significantly reduced in BC-/EC+ (5min n=4 and 24h n=5) compared to BC+/EC+ (5min n=4 ; *p<0.05 and 24h n=6; *p<0.05) at 5 minutes and 24 hours of reperfusion. Nitrite and nitrate concentration in heart tissue did not differ between both groups at 5 min (nitrite, n=4 per group, n.s.; nitrate: BC+/EC n=3, BC-/EC+ n=4, n.s) and 24 hours of reperfusion (nitrite, n=3 per group, n.s.; nitrate, n=3 per group; n.s.; 2-way ANOVA followed by Bonferroni´s post hoc test).
Methods Online Resource 5 – Measurement of nitrite/nitrate in plasma and heart tissue
(Methods description included in main manuscript, reproduced here for convenience)
Blood samples were collected by intra-cardiac puncture at baseline, after 5 Min of reperfusion and 24h of myocardial reperfusion. Blood and tissue samples were prepared for determination of nitrate and nitrite (Rassaf, Feelisch et al. 2004). Blood samples were centrifuged and plasma was aspirated and stored at -80 °C. To perform analysis, frozen plasma was thawed and mixed with methanol (1:1, v/v) to precipitate the proteins. Samples were centrifuged again and a NOx-analyzing system (ENO-20 Analyzer, EICOM Corp., Kyoto, Japan) was used to analyze the supernatant..
After thoracotomy, a cannula was inserted into the heart and the heart was flushed free of blood by in situ perfusion with NaCl at a rate of 10 ml/min. After perfusion, hearts were excised from the mice, snap frozen in liquid nitrogen and stored at -80 °C. To perform analysis, frozen hearts were homogenized immediately in ice-cold NaCl by using a Schuett homogenplus semi-automatic glass-on-glass-homogenizer and kept on ice and mixed with methanol (1:1, v/v) to precipitate the proteins. Samples were centrifuged again and a NOx-analyzing system (ENO-20 Analyzer, EICOM Corp., Kyoto, Japan) was used to analyze the supernatant. The high-pressure liquid chromatography (HPLC) technique is a highly sensitive technique for measurement of nitrite and nitrate. This method employs ion chromatography with online reduction of nitrate to nitrite and subsequent postcolumn derivatization with the Griess reagent. The detection limit for nitrite and nitrate is 1 nmol/l for either anion at an injection volume of 100 µl (Rassaf, Bryan et al. 2002; Kleinbongard, Dejam et al. 2003; Grau, Hendgen-Cotta et al. 2007; Hendgen-Cotta, Grau et al. 2008).
