# Supplementary material for: Depletion of circulating blood NOS3 increases severity of myocardial infarction and left ventricular dysfunction
Source: Basic Res Cardiol. 2013 Dec 18;109(1):398. doi: 10.1007/s00395-013-0398-1 (PMC3898535; doi:10.1007/s00395-013-0398-1)
Supplement: Supplementary file 6 — Supplementary material 6 (PPTX 72 kb) [file 395_2013_398_MOESM6_ESM.pptx]

## Slide 1
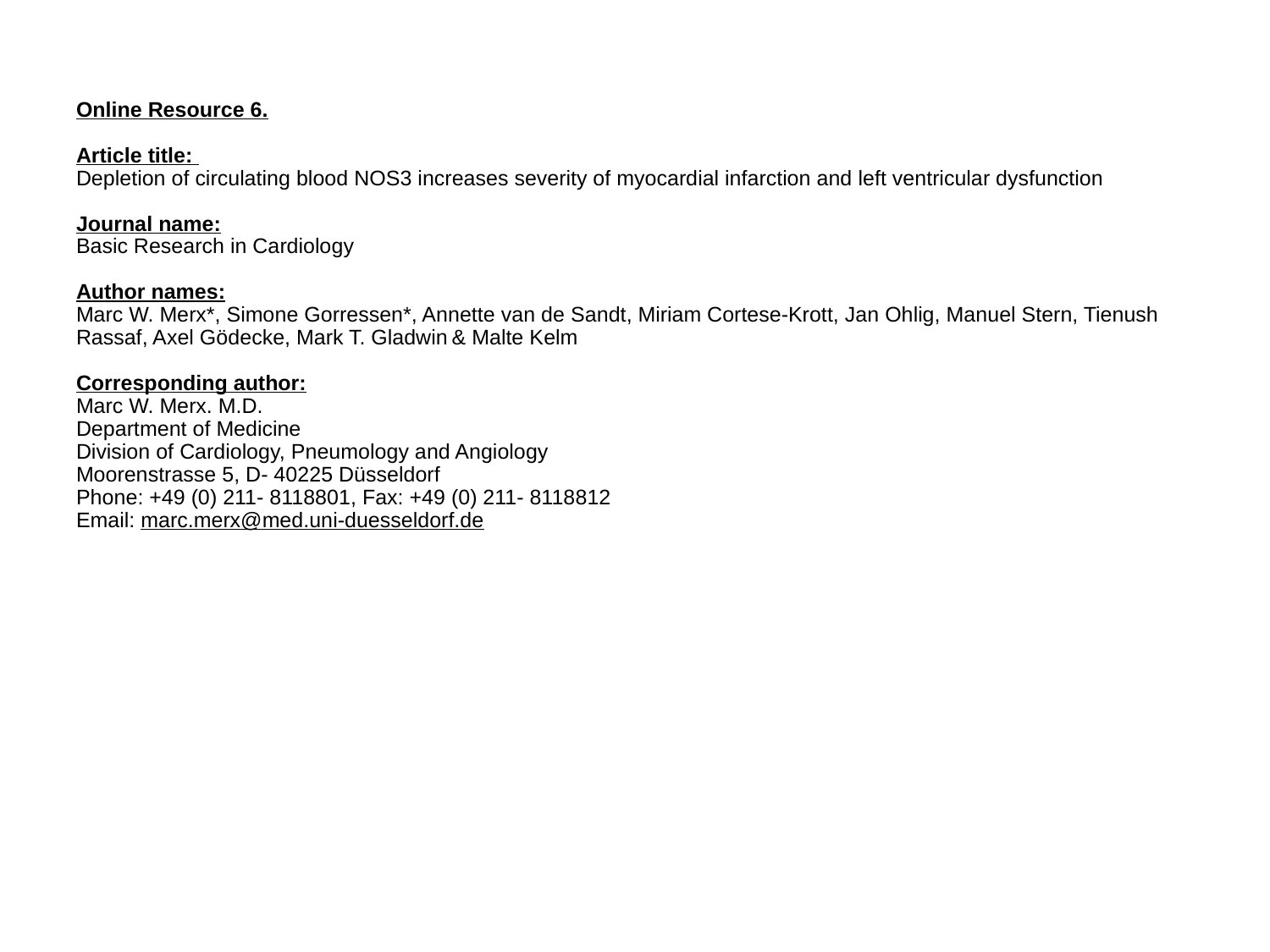

# Online Resource 6. Article title: Depletion of circulating blood NOS3 increases severity of myocardial infarction and left ventricular dysfunction Journal name: Basic Research in Cardiology Author names: Marc W. Merx*, Simone Gorressen*, Annette van de Sandt, Miriam Cortese-Krott, Jan Ohlig, Manuel Stern, Tienush Rassaf, Axel Gödecke, Mark T. Gladwin & Malte Kelm Corresponding author: Marc W. Merx. M.D. Department of Medicine Division of Cardiology, Pneumology and Angiology Moorenstrasse 5, D- 40225 Düsseldorf Phone: +49 (0) 211- 8118801, Fax: +49 (0) 211- 8118812 Email: marc.merx@med.uni-duesseldorf.de

## Slide 2
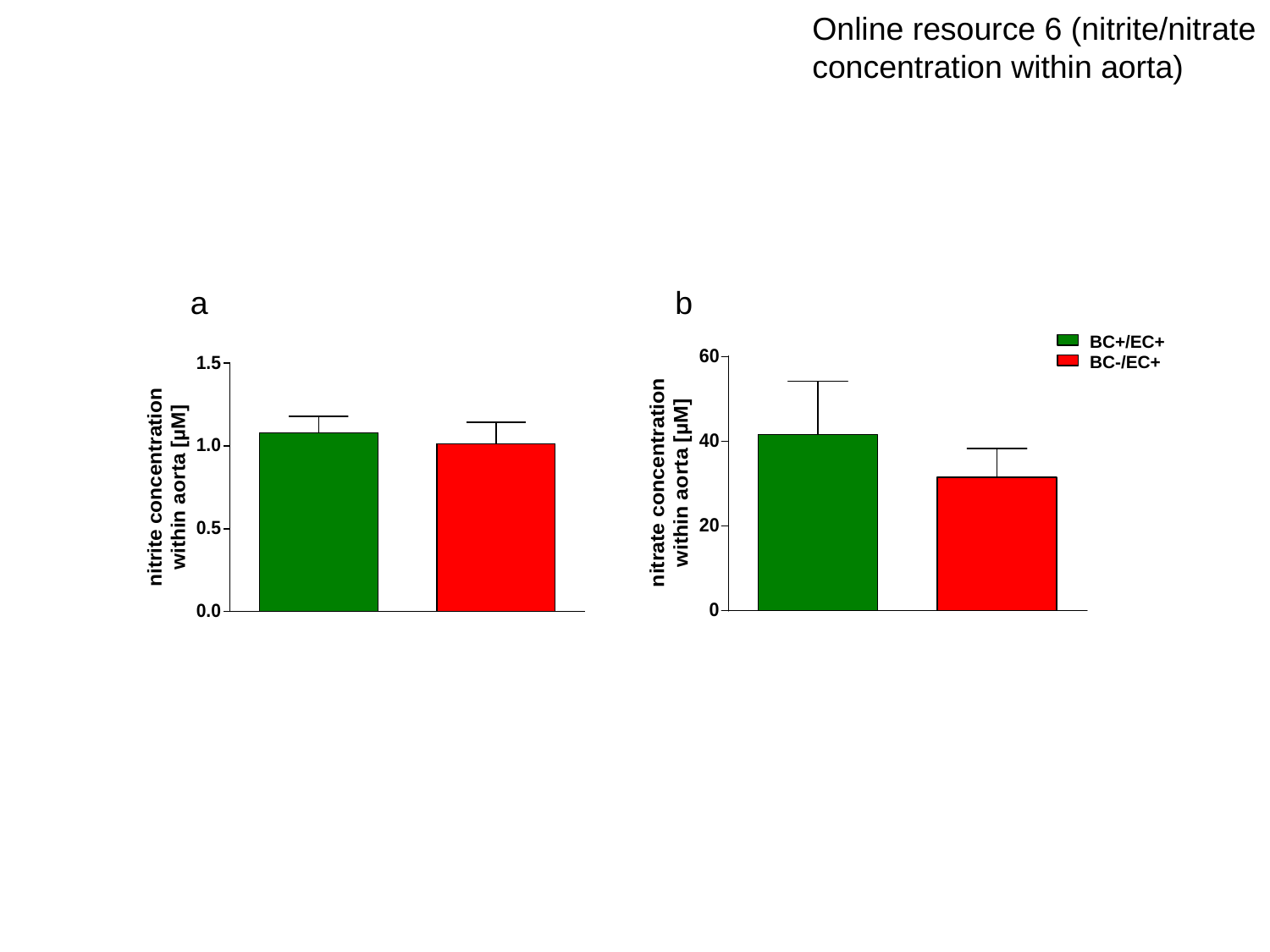

Online resource 6 (nitrite/nitrate
concentration within aorta)
a
b
BC+/EC+
BC-/EC+

## Slide 3
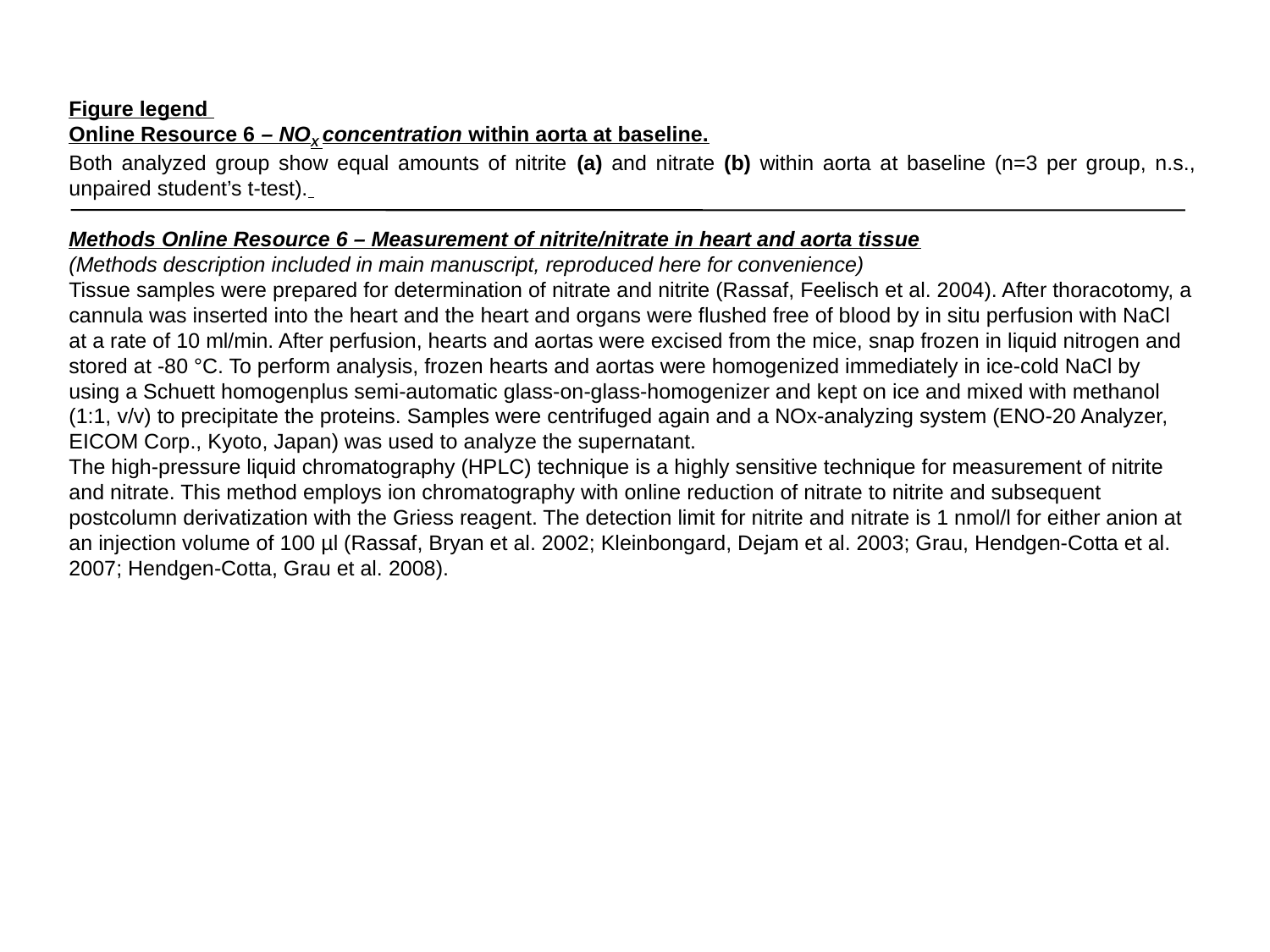

Figure legend
Online Resource 6 – NOX concentration within aorta at baseline.
Both analyzed group show equal amounts of nitrite (a) and nitrate (b) within aorta at baseline (n=3 per group, n.s., unpaired student’s t-test).
Methods Online Resource 6 – Measurement of nitrite/nitrate in heart and aorta tissue
(Methods description included in main manuscript, reproduced here for convenience)
Tissue samples were prepared for determination of nitrate and nitrite (Rassaf, Feelisch et al. 2004). After thoracotomy, a cannula was inserted into the heart and the heart and organs were flushed free of blood by in situ perfusion with NaCl at a rate of 10 ml/min. After perfusion, hearts and aortas were excised from the mice, snap frozen in liquid nitrogen and stored at -80 °C. To perform analysis, frozen hearts and aortas were homogenized immediately in ice-cold NaCl by using a Schuett homogenplus semi-automatic glass-on-glass-homogenizer and kept on ice and mixed with methanol (1:1, v/v) to precipitate the proteins. Samples were centrifuged again and a NOx-analyzing system (ENO-20 Analyzer, EICOM Corp., Kyoto, Japan) was used to analyze the supernatant.
The high-pressure liquid chromatography (HPLC) technique is a highly sensitive technique for measurement of nitrite and nitrate. This method employs ion chromatography with online reduction of nitrate to nitrite and subsequent postcolumn derivatization with the Griess reagent. The detection limit for nitrite and nitrate is 1 nmol/l for either anion at an injection volume of 100 µl (Rassaf, Bryan et al. 2002; Kleinbongard, Dejam et al. 2003; Grau, Hendgen-Cotta et al. 2007; Hendgen-Cotta, Grau et al. 2008).
