# Supplementary material for: Depletion of circulating blood NOS3 increases severity of myocardial infarction and left ventricular dysfunction
Source: Basic Res Cardiol. 2013 Dec 18;109(1):398. doi: 10.1007/s00395-013-0398-1 (PMC3898535; doi:10.1007/s00395-013-0398-1)
Supplement: Supplementary file 7 — Supplementary material 7 (PPTX 70 kb) [file 395_2013_398_MOESM7_ESM.pptx]

## Slide 1
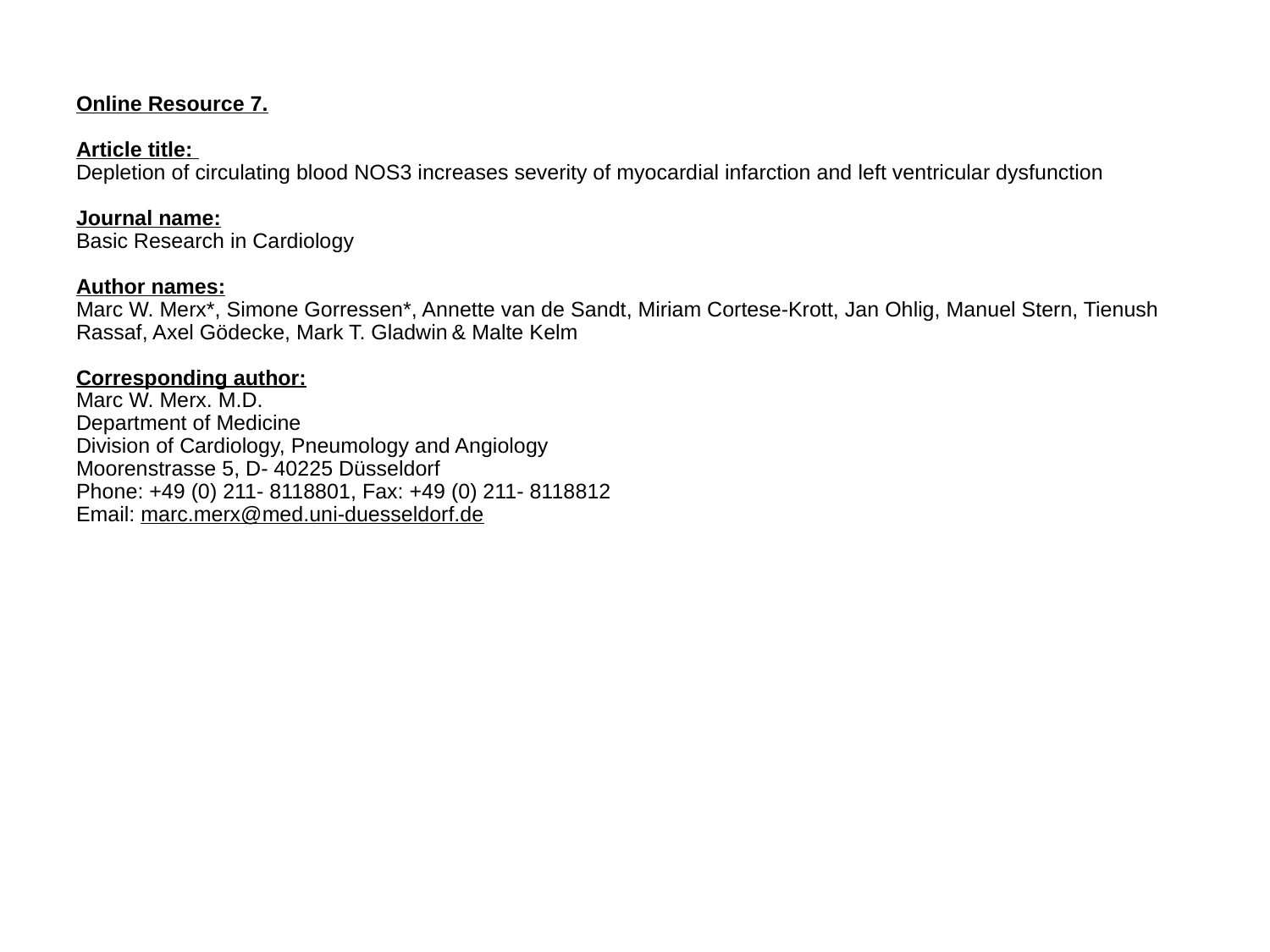

# Online Resource 7. Article title: Depletion of circulating blood NOS3 increases severity of myocardial infarction and left ventricular dysfunction Journal name: Basic Research in Cardiology Author names: Marc W. Merx*, Simone Gorressen*, Annette van de Sandt, Miriam Cortese-Krott, Jan Ohlig, Manuel Stern, Tienush Rassaf, Axel Gödecke, Mark T. Gladwin & Malte Kelm Corresponding author: Marc W. Merx. M.D. Department of Medicine Division of Cardiology, Pneumology and Angiology Moorenstrasse 5, D- 40225 Düsseldorf Phone: +49 (0) 211- 8118801, Fax: +49 (0) 211- 8118812 Email: marc.merx@med.uni-duesseldorf.de

## Slide 2
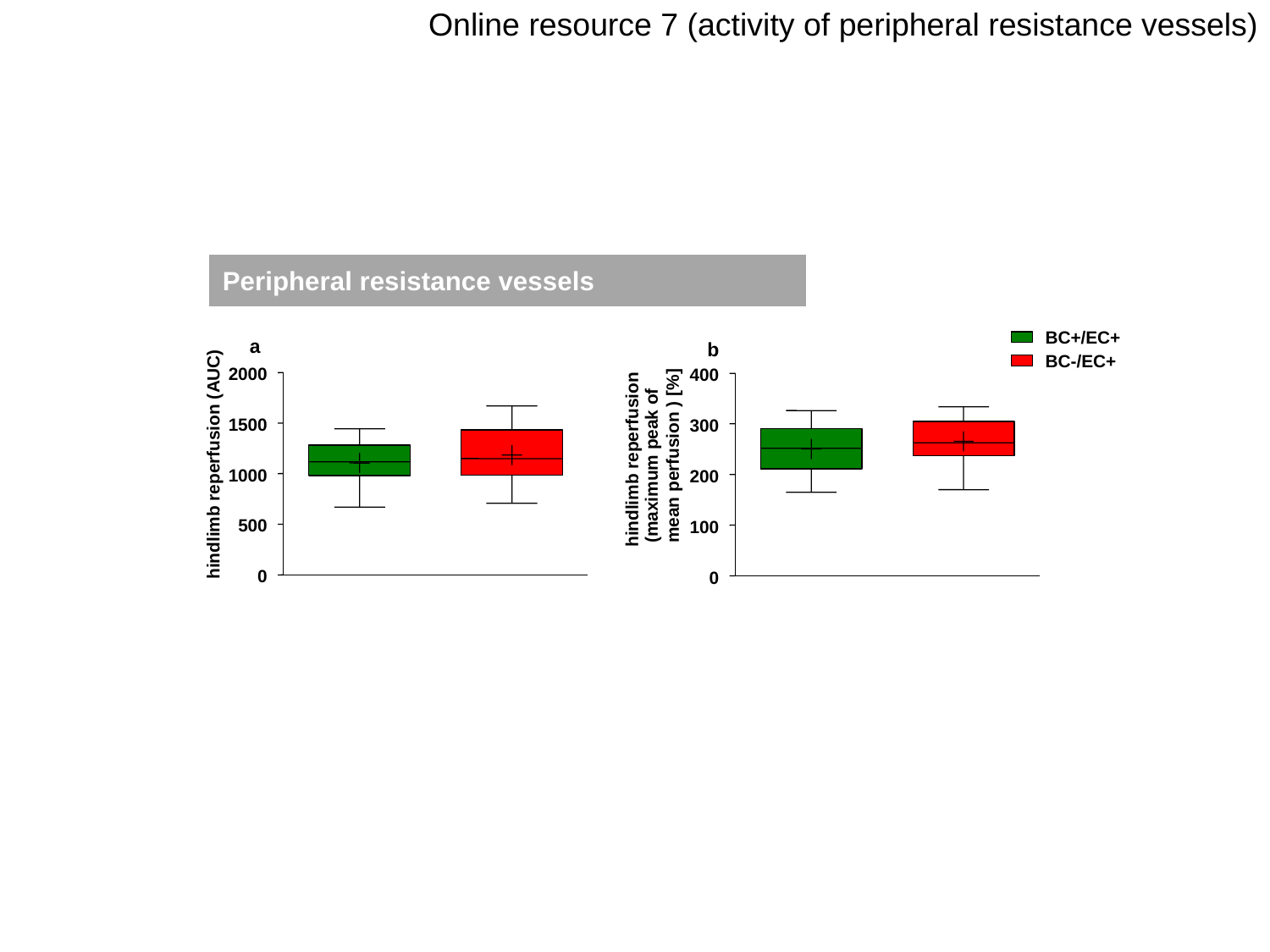

Online resource 7 (activity of peripheral resistance vessels)
Peripheral resistance vessels
a
2000
1500
hindlimb reperfusion (AUC)
1000
500
0
BC+/EC+
b
BC-/EC+
400
300
(maximum peak of
mean perfusion ) [%]
hindlimb reperfusion
200
100
0

## Slide 3
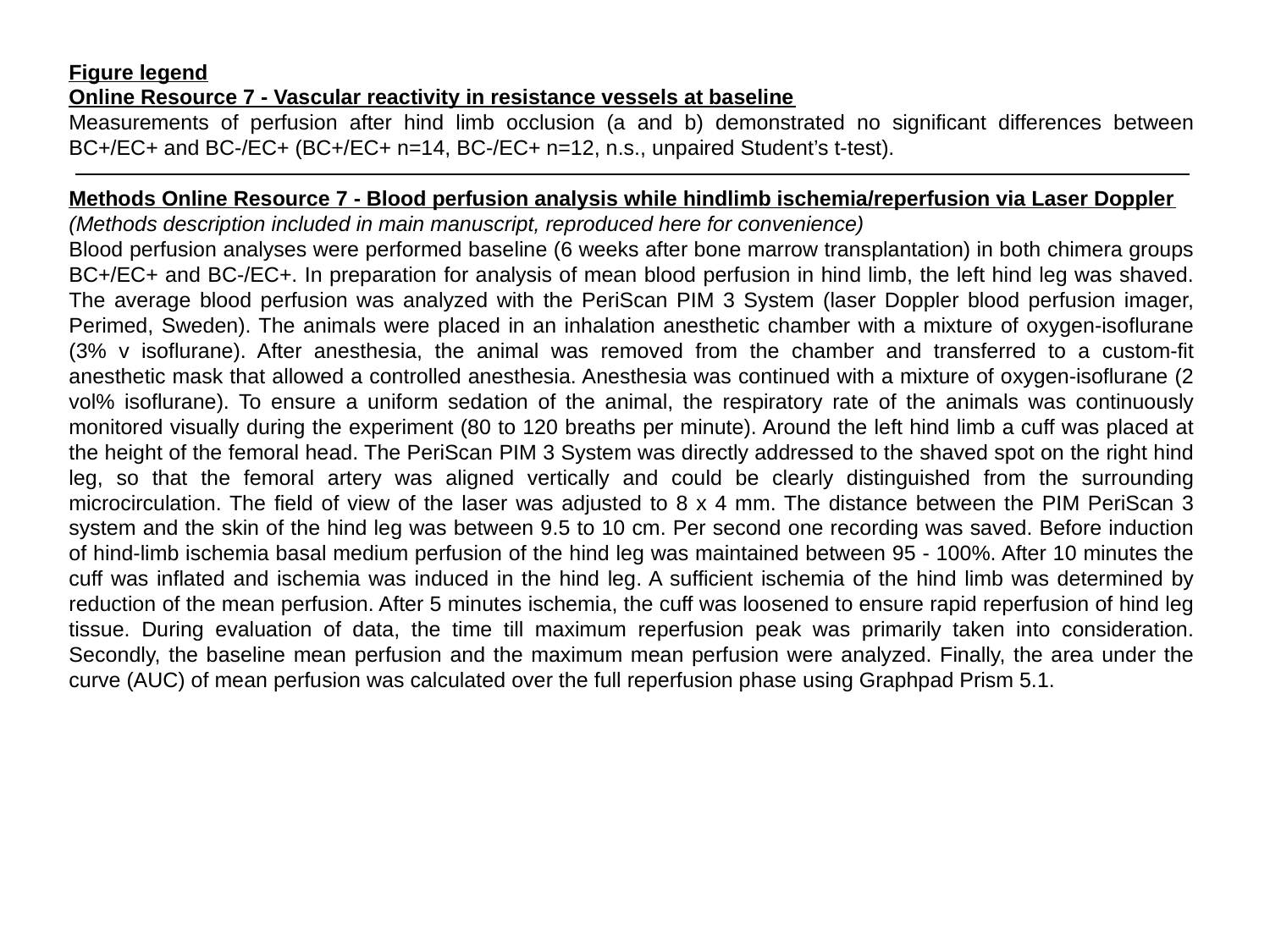

Figure legend
Online Resource 7 - Vascular reactivity in resistance vessels at baseline
Measurements of perfusion after hind limb occlusion (a and b) demonstrated no significant differences between BC+/EC+ and BC-/EC+ (BC+/EC+ n=14, BC-/EC+ n=12, n.s., unpaired Student’s t-test).
Methods Online Resource 7 - Blood perfusion analysis while hindlimb ischemia/reperfusion via Laser Doppler
(Methods description included in main manuscript, reproduced here for convenience)
Blood perfusion analyses were performed baseline (6 weeks after bone marrow transplantation) in both chimera groups BC+/EC+ and BC-/EC+. In preparation for analysis of mean blood perfusion in hind limb, the left hind leg was shaved. The average blood perfusion was analyzed with the PeriScan PIM 3 System (laser Doppler blood perfusion imager, Perimed, Sweden). The animals were placed in an inhalation anesthetic chamber with a mixture of oxygen-isoflurane (3% v isoflurane). After anesthesia, the animal was removed from the chamber and transferred to a custom-fit anesthetic mask that allowed a controlled anesthesia. Anesthesia was continued with a mixture of oxygen-isoflurane (2 vol% isoflurane). To ensure a uniform sedation of the animal, the respiratory rate of the animals was continuously monitored visually during the experiment (80 to 120 breaths per minute). Around the left hind limb a cuff was placed at the height of the femoral head. The PeriScan PIM 3 System was directly addressed to the shaved spot on the right hind leg, so that the femoral artery was aligned vertically and could be clearly distinguished from the surrounding microcirculation. The field of view of the laser was adjusted to 8 x 4 mm. The distance between the PIM PeriScan 3 system and the skin of the hind leg was between 9.5 to 10 cm. Per second one recording was saved. Before induction of hind-limb ischemia basal medium perfusion of the hind leg was maintained between 95 - 100%. After 10 minutes the cuff was inflated and ischemia was induced in the hind leg. A sufficient ischemia of the hind limb was determined by reduction of the mean perfusion. After 5 minutes ischemia, the cuff was loosened to ensure rapid reperfusion of hind leg tissue. During evaluation of data, the time till maximum reperfusion peak was primarily taken into consideration. Secondly, the baseline mean perfusion and the maximum mean perfusion were analyzed. Finally, the area under the curve (AUC) of mean perfusion was calculated over the full reperfusion phase using Graphpad Prism 5.1.
