# Supplementary material for: Depletion of circulating blood NOS3 increases severity of myocardial infarction and left ventricular dysfunction
Source: Basic Res Cardiol. 2013 Dec 18;109(1):398. doi: 10.1007/s00395-013-0398-1 (PMC3898535; doi:10.1007/s00395-013-0398-1)
Supplement: Supplementary file 8 — Supplementary material 8 (PPTX 63 kb) [file 395_2013_398_MOESM8_ESM.pptx]

## Slide 1
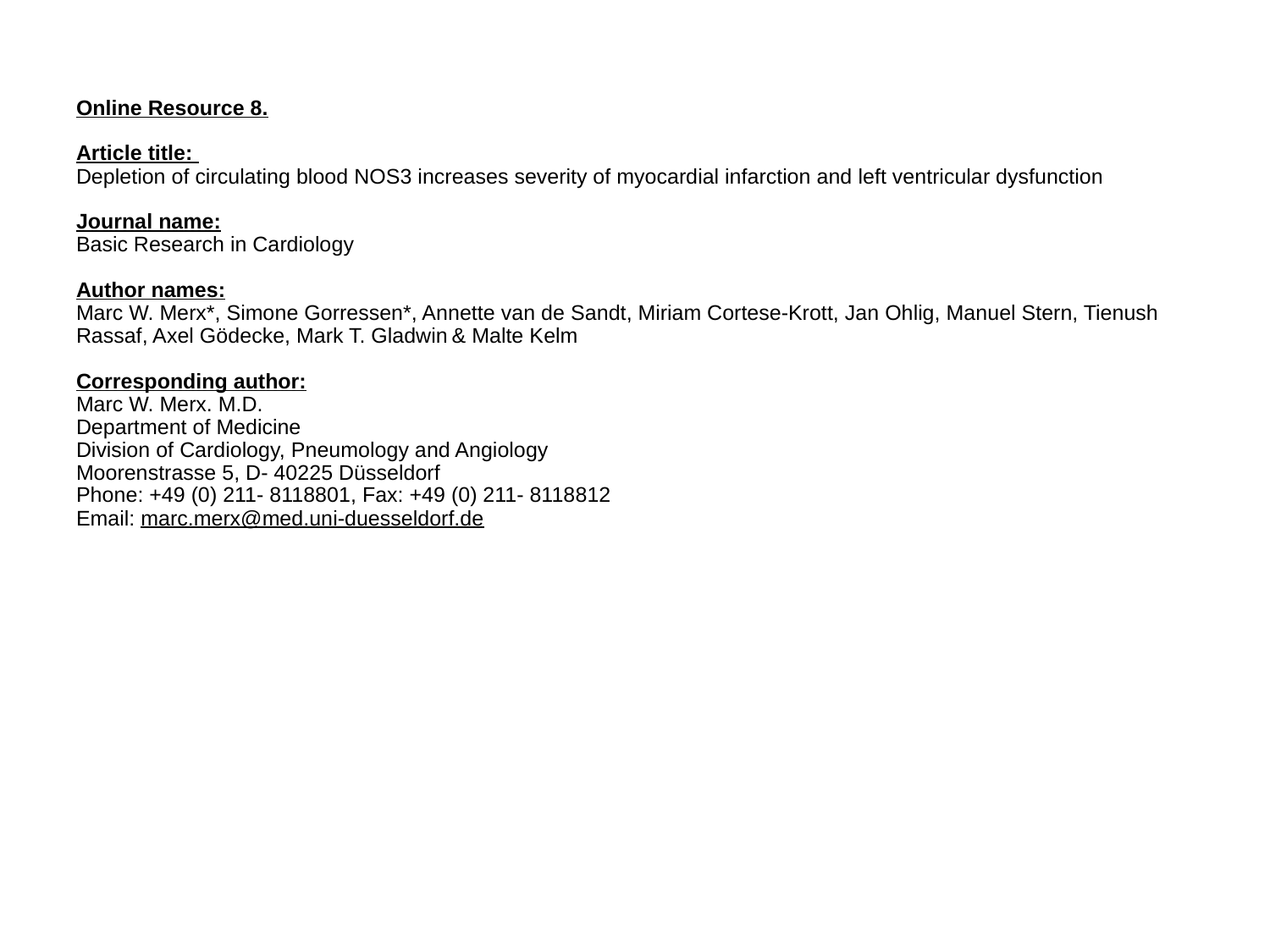

# Online Resource 8. Article title: Depletion of circulating blood NOS3 increases severity of myocardial infarction and left ventricular dysfunction Journal name: Basic Research in Cardiology Author names: Marc W. Merx*, Simone Gorressen*, Annette van de Sandt, Miriam Cortese-Krott, Jan Ohlig, Manuel Stern, Tienush Rassaf, Axel Gödecke, Mark T. Gladwin & Malte Kelm Corresponding author: Marc W. Merx. M.D. Department of Medicine Division of Cardiology, Pneumology and Angiology Moorenstrasse 5, D- 40225 Düsseldorf Phone: +49 (0) 211- 8118801, Fax: +49 (0) 211- 8118812 Email: marc.merx@med.uni-duesseldorf.de

## Slide 2
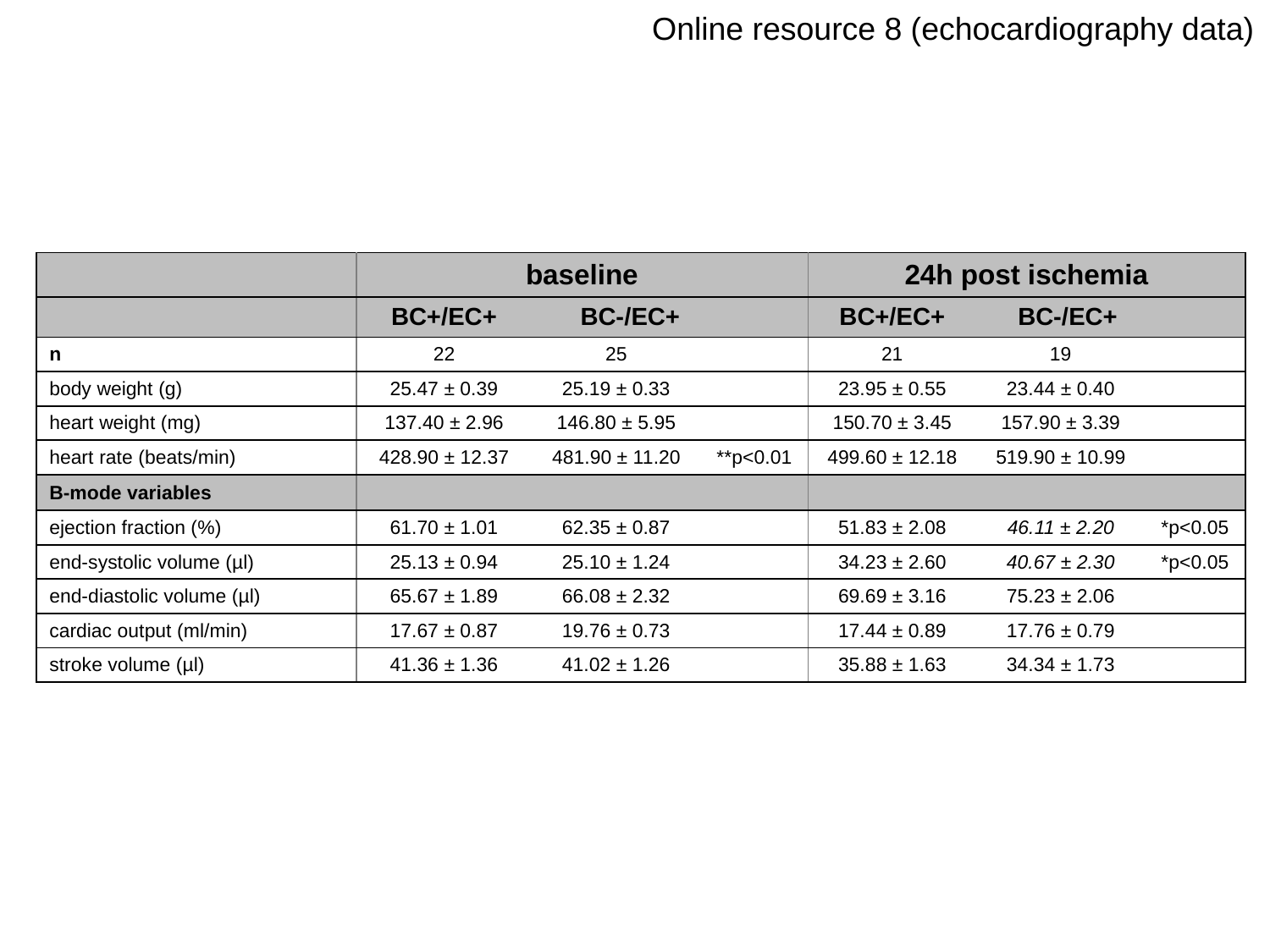

Online resource 8 (echocardiography data)
| | baseline | | | 24h post ischemia | | |
| --- | --- | --- | --- | --- | --- | --- |
| | BC+/EC+ | BC-/EC+ | | BC+/EC+ | BC-/EC+ | |
| n | 22 | 25 | | 21 | 19 | |
| body weight (g) | 25.47 ± 0.39 | 25.19 ± 0.33 | | 23.95 ± 0.55 | 23.44 ± 0.40 | |
| heart weight (mg) | 137.40 ± 2.96 | 146.80 ± 5.95 | | 150.70 ± 3.45 | 157.90 ± 3.39 | |
| heart rate (beats/min) | 428.90 ± 12.37 | 481.90 ± 11.20 | \*\*p<0.01 | 499.60 ± 12.18 | 519.90 ± 10.99 | |
| B-mode variables | | | | | | |
| ejection fraction (%) | 61.70 ± 1.01 | 62.35 ± 0.87 | | 51.83 ± 2.08 | 46.11 ± 2.20 | \*p<0.05 |
| end-systolic volume (µl) | 25.13 ± 0.94 | 25.10 ± 1.24 | | 34.23 ± 2.60 | 40.67 ± 2.30 | \*p<0.05 |
| end-diastolic volume (µl) | 65.67 ± 1.89 | 66.08 ± 2.32 | | 69.69 ± 3.16 | 75.23 ± 2.06 | |
| cardiac output (ml/min) | 17.67 ± 0.87 | 19.76 ± 0.73 | | 17.44 ± 0.89 | 17.76 ± 0.79 | |
| stroke volume (µl) | 41.36 ± 1.36 | 41.02 ± 1.26 | | 35.88 ± 1.63 | 34.34 ± 1.73 | |

## Slide 3
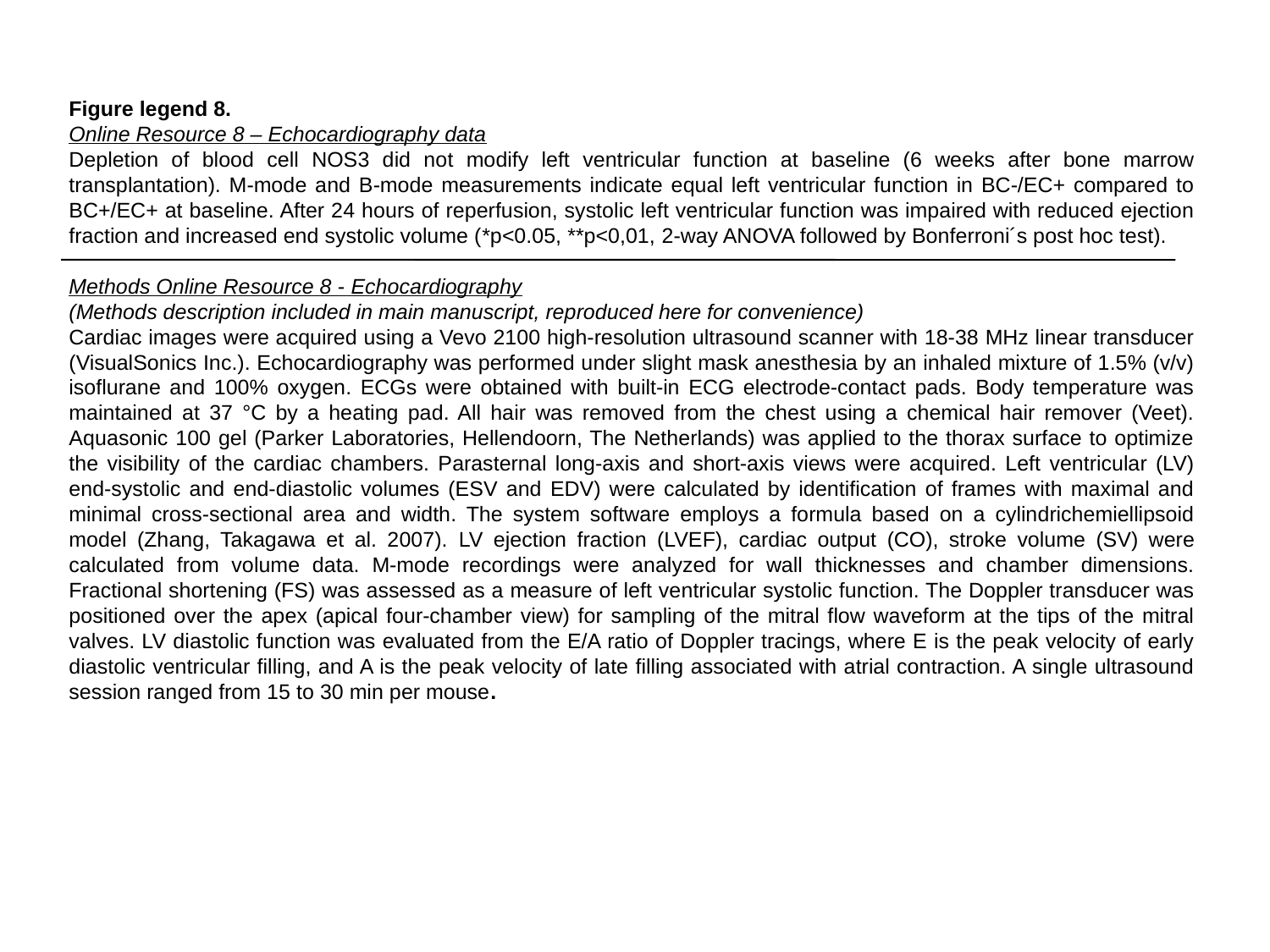

Figure legend 8.
Online Resource 8 – Echocardiography data
Depletion of blood cell NOS3 did not modify left ventricular function at baseline (6 weeks after bone marrow transplantation). M-mode and B-mode measurements indicate equal left ventricular function in BC-/EC+ compared to BC+/EC+ at baseline. After 24 hours of reperfusion, systolic left ventricular function was impaired with reduced ejection fraction and increased end systolic volume (*p<0.05, **p<0,01, 2-way ANOVA followed by Bonferroni´s post hoc test).
Methods Online Resource 8 - Echocardiography
(Methods description included in main manuscript, reproduced here for convenience)
Cardiac images were acquired using a Vevo 2100 high-resolution ultrasound scanner with 18-38 MHz linear transducer (VisualSonics Inc.). Echocardiography was performed under slight mask anesthesia by an inhaled mixture of 1.5% (v/v) isoflurane and 100% oxygen. ECGs were obtained with built-in ECG electrode-contact pads. Body temperature was maintained at 37 °C by a heating pad. All hair was removed from the chest using a chemical hair remover (Veet). Aquasonic 100 gel (Parker Laboratories, Hellendoorn, The Netherlands) was applied to the thorax surface to optimize the visibility of the cardiac chambers. Parasternal long-axis and short-axis views were acquired. Left ventricular (LV) end-systolic and end-diastolic volumes (ESV and EDV) were calculated by identification of frames with maximal and minimal cross-sectional area and width. The system software employs a formula based on a cylindrichemiellipsoid model (Zhang, Takagawa et al. 2007). LV ejection fraction (LVEF), cardiac output (CO), stroke volume (SV) were calculated from volume data. M-mode recordings were analyzed for wall thicknesses and chamber dimensions. Fractional shortening (FS) was assessed as a measure of left ventricular systolic function. The Doppler transducer was positioned over the apex (apical four-chamber view) for sampling of the mitral flow waveform at the tips of the mitral valves. LV diastolic function was evaluated from the E/A ratio of Doppler tracings, where E is the peak velocity of early diastolic ventricular filling, and A is the peak velocity of late filling associated with atrial contraction. A single ultrasound session ranged from 15 to 30 min per mouse.
