# Supplementary material for: Depletion of circulating blood NOS3 increases severity of myocardial infarction and left ventricular dysfunction
Source: Basic Res Cardiol. 2013 Dec 18;109(1):398. doi: 10.1007/s00395-013-0398-1 (PMC3898535; doi:10.1007/s00395-013-0398-1)
Supplement: Supplementary file 9 — Supplementary material 9 (PPTX 66 kb) [file 395_2013_398_MOESM9_ESM.pptx]

## Slide 1
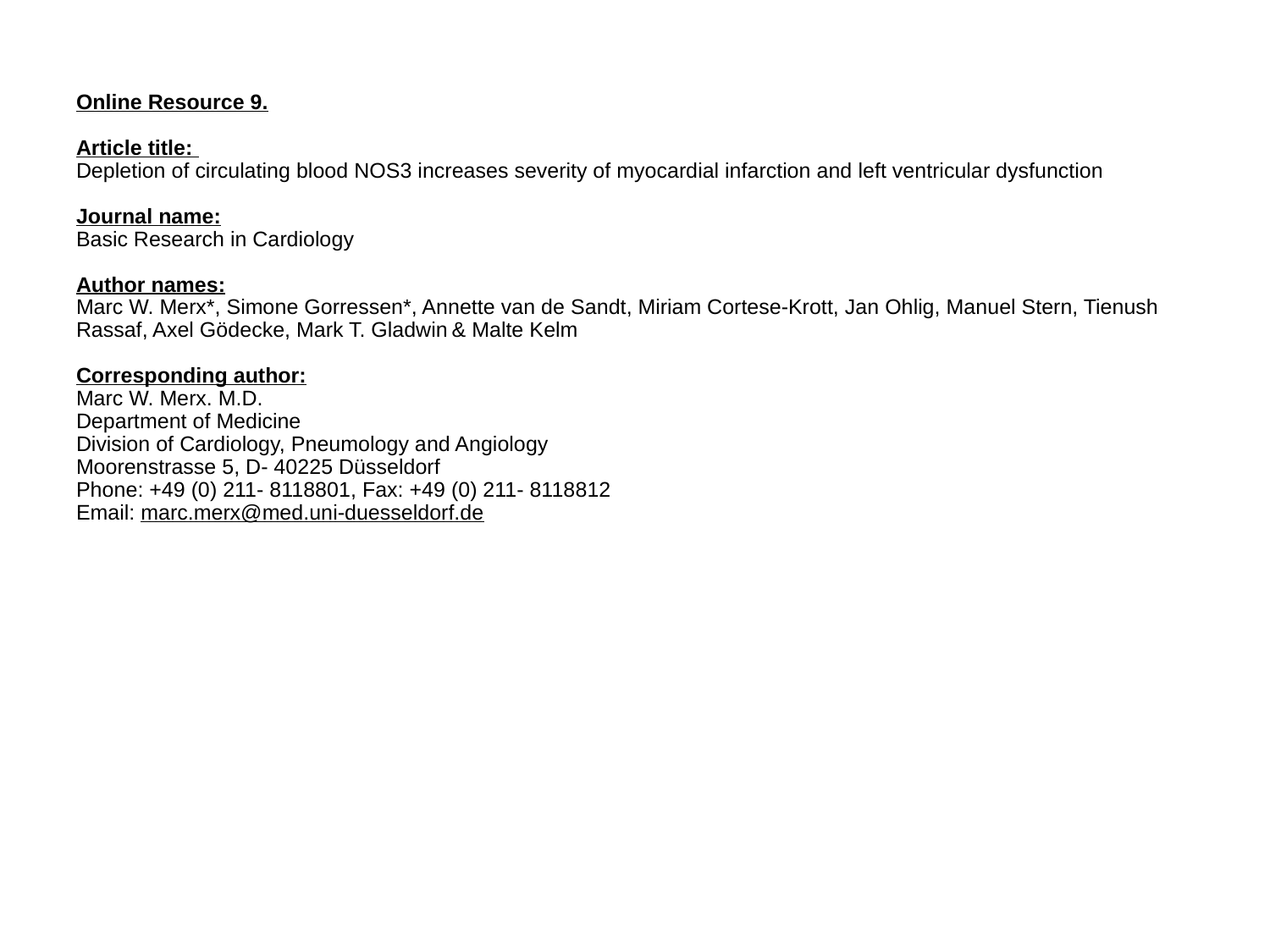

# Online Resource 9. Article title: Depletion of circulating blood NOS3 increases severity of myocardial infarction and left ventricular dysfunction Journal name: Basic Research in Cardiology Author names: Marc W. Merx*, Simone Gorressen*, Annette van de Sandt, Miriam Cortese-Krott, Jan Ohlig, Manuel Stern, Tienush Rassaf, Axel Gödecke, Mark T. Gladwin & Malte Kelm Corresponding author: Marc W. Merx. M.D. Department of Medicine Division of Cardiology, Pneumology and Angiology Moorenstrasse 5, D- 40225 Düsseldorf Phone: +49 (0) 211- 8118801, Fax: +49 (0) 211- 8118812 Email: marc.merx@med.uni-duesseldorf.de

## Slide 2
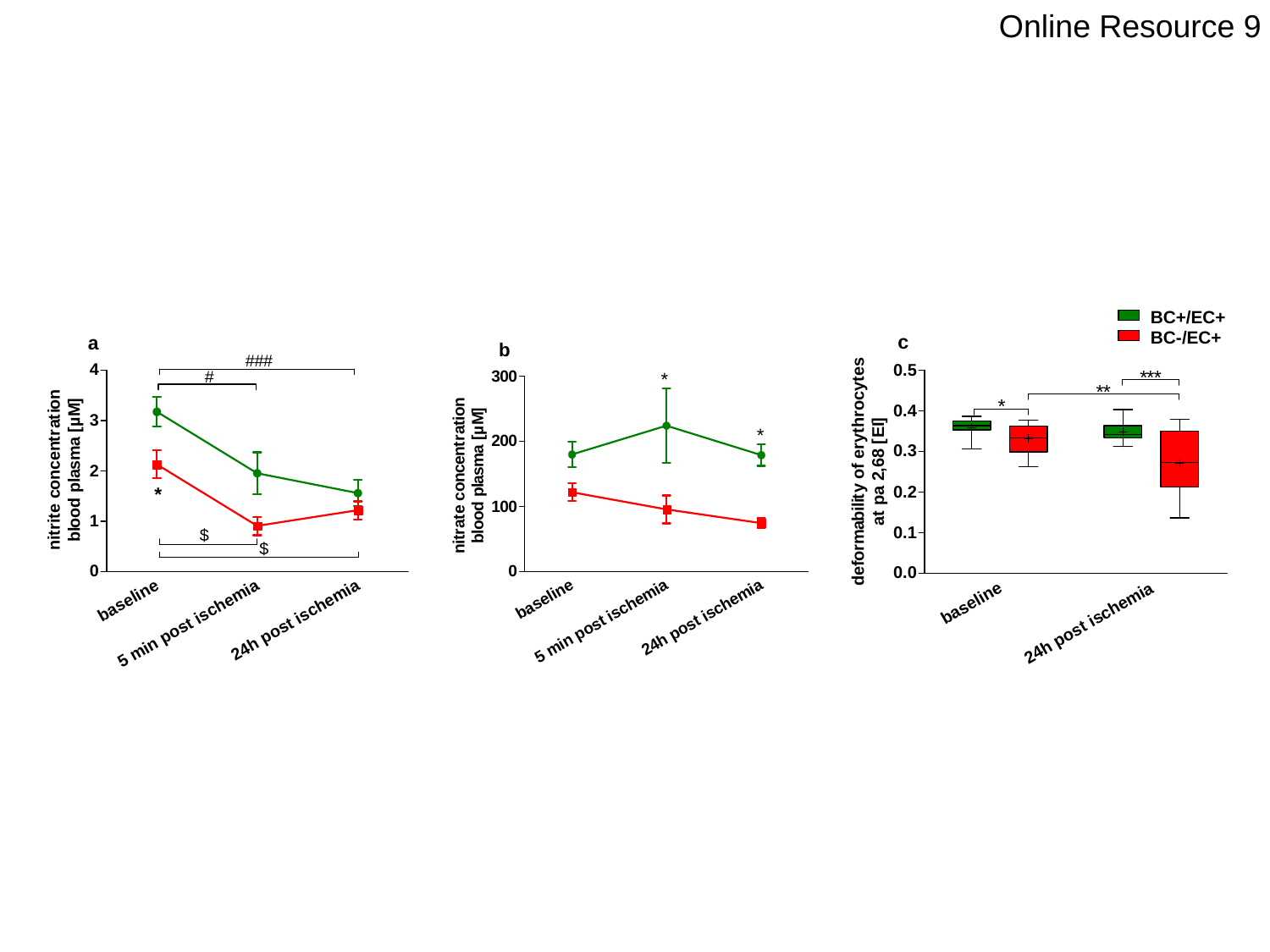

Online Resource 9
BC+/EC+
BC-/EC+

## Slide 3
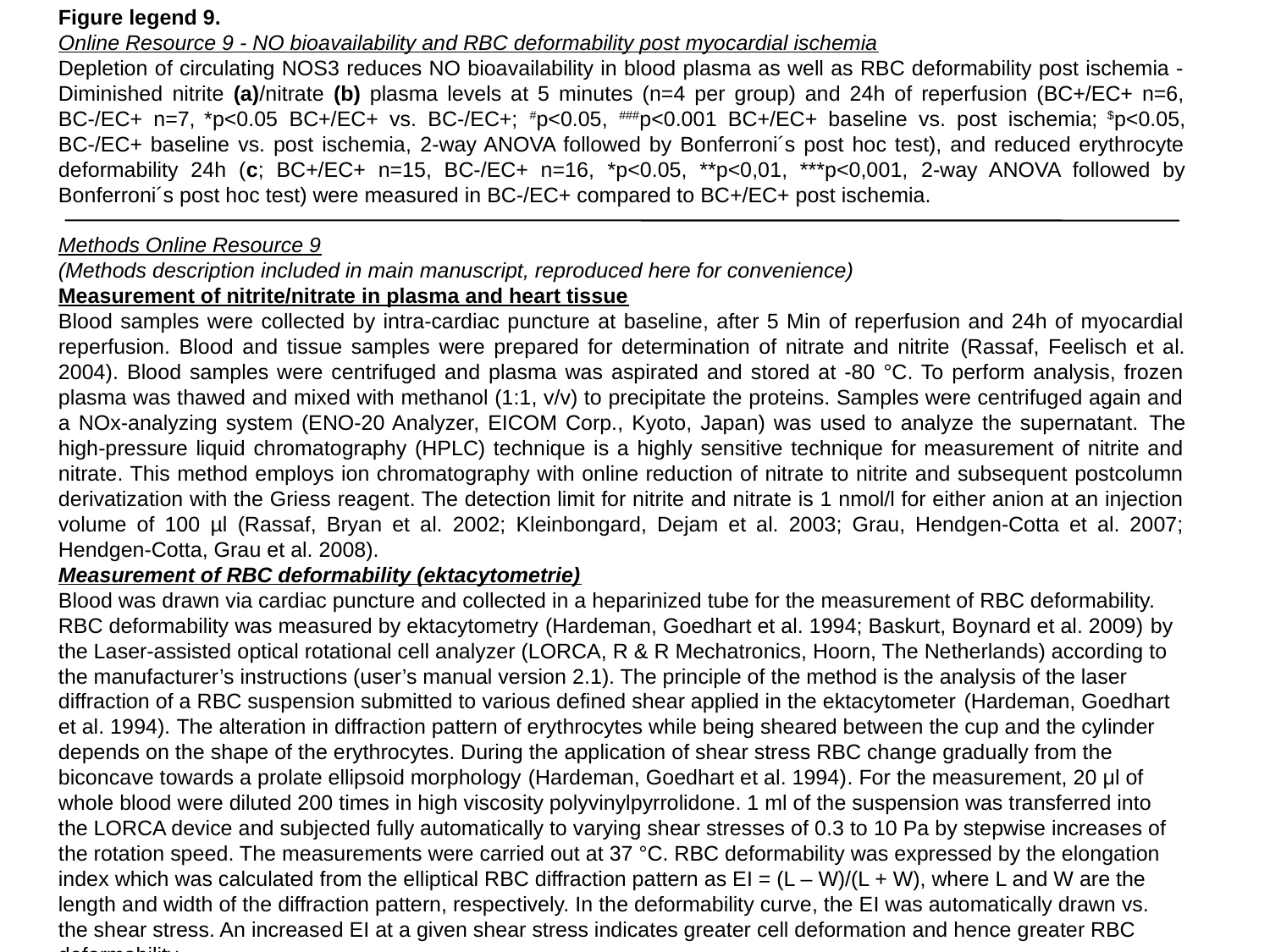

Figure legend 9.
Online Resource 9 - NO bioavailability and RBC deformability post myocardial ischemia
Depletion of circulating NOS3 reduces NO bioavailability in blood plasma as well as RBC deformability post ischemia - Diminished nitrite (a)/nitrate (b) plasma levels at 5 minutes (n=4 per group) and 24h of reperfusion (BC+/EC+ n=6, BC-/EC+ n=7, *p<0.05 BC+/EC+ vs. BC-/EC+; #p<0.05, ###p<0.001 BC+/EC+ baseline vs. post ischemia; $p<0.05, BC-/EC+ baseline vs. post ischemia, 2-way ANOVA followed by Bonferroni´s post hoc test), and reduced erythrocyte deformability 24h (c; BC+/EC+ n=15, BC-/EC+ n=16, *p<0.05, **p<0,01, ***p<0,001, 2-way ANOVA followed by Bonferroni´s post hoc test) were measured in BC-/EC+ compared to BC+/EC+ post ischemia.
Methods Online Resource 9
(Methods description included in main manuscript, reproduced here for convenience)
Measurement of nitrite/nitrate in plasma and heart tissue
Blood samples were collected by intra-cardiac puncture at baseline, after 5 Min of reperfusion and 24h of myocardial reperfusion. Blood and tissue samples were prepared for determination of nitrate and nitrite (Rassaf, Feelisch et al. 2004). Blood samples were centrifuged and plasma was aspirated and stored at -80 °C. To perform analysis, frozen plasma was thawed and mixed with methanol (1:1, v/v) to precipitate the proteins. Samples were centrifuged again and a NOx-analyzing system (ENO-20 Analyzer, EICOM Corp., Kyoto, Japan) was used to analyze the supernatant. The high-pressure liquid chromatography (HPLC) technique is a highly sensitive technique for measurement of nitrite and nitrate. This method employs ion chromatography with online reduction of nitrate to nitrite and subsequent postcolumn derivatization with the Griess reagent. The detection limit for nitrite and nitrate is 1 nmol/l for either anion at an injection volume of 100 µl (Rassaf, Bryan et al. 2002; Kleinbongard, Dejam et al. 2003; Grau, Hendgen-Cotta et al. 2007; Hendgen-Cotta, Grau et al. 2008).
Measurement of RBC deformability (ektacytometrie)
Blood was drawn via cardiac puncture and collected in a heparinized tube for the measurement of RBC deformability. RBC deformability was measured by ektacytometry (Hardeman, Goedhart et al. 1994; Baskurt, Boynard et al. 2009) by the Laser-assisted optical rotational cell analyzer (LORCA, R & R Mechatronics, Hoorn, The Netherlands) according to the manufacturer’s instructions (user’s manual version 2.1). The principle of the method is the analysis of the laser diffraction of a RBC suspension submitted to various defined shear applied in the ektacytometer (Hardeman, Goedhart et al. 1994). The alteration in diffraction pattern of erythrocytes while being sheared between the cup and the cylinder depends on the shape of the erythrocytes. During the application of shear stress RBC change gradually from the biconcave towards a prolate ellipsoid morphology (Hardeman, Goedhart et al. 1994). For the measurement, 20 μl of whole blood were diluted 200 times in high viscosity polyvinylpyrrolidone. 1 ml of the suspension was transferred into the LORCA device and subjected fully automatically to varying shear stresses of 0.3 to 10 Pa by stepwise increases of the rotation speed. The measurements were carried out at 37 °C. RBC deformability was expressed by the elongation index which was calculated from the elliptical RBC diffraction pattern as EI = (L – W)/(L + W), where L and W are the length and width of the diffraction pattern, respectively. In the deformability curve, the EI was automatically drawn vs. the shear stress. An increased EI at a given shear stress indicates greater cell deformation and hence greater RBC deformability.
